# Beta secretase 1-dependent amyloid precursor protein processing promotes excessive vascular sprouting through NOTCH3 signalling

**DOI:** 10.1038/s41419-020-2288-4

**Published:** 2020-02-06

**Authors:** Claire S. Durrant, Karsten Ruscher, Olivia Sheppard, Michael P. Coleman, Ilknur Özen

**Affiliations:** 1 John van Geest Centre for Brain Repair, Forvie Site, Robinson Way, Cambridge, CB2 0PY UK; 2 The Babraham Institute, Babraham Research Campus, Cambridge, CB22 3AT UK; 3 Centre for Discovery Brain Sciences, University of Edinburgh, 1 George Square, Edinburgh, EH8 9JZ UK; 4 Laboratory for Experimental Brain Research, Department of Clinical Sciences, Lund University, SE, Lund, Sweden; 5 Lund Brain Injury Laboratory for Neurosurgical Research, Department of Clinical Sciences, Lund University, Lund, Sweden; 6 Present Address: John van Geest Centre for Brain Repair, Forvie Site, Robinson Way, Cambridge, CB2 0PY UK; 7 Present Address: Lund Brain Injury Laboratory for Neurosurgical Research, Wallenberg Neuroscience Center, Lund University, BMC A13, 221 84 Lund, Sweden

**Keywords:** Cellular neuroscience, Alzheimer's disease

## Abstract

Amyloid beta peptides (Aβ) proteins play a key role in vascular pathology in Alzheimer’s Disease (AD) including impairment of the blood–brain barrier and aberrant angiogenesis. Although previous work has demonstrated a pro-angiogenic role of Aβ, the exact mechanisms by which amyloid precursor protein (APP) processing and endothelial angiogenic signalling cascades interact in AD remain a largely unsolved problem. Here, we report that increased endothelial sprouting in human-APP transgenic mouse (TgCRND8) tissue is dependent on β-secretase (BACE1) processing of APP. Higher levels of Aβ processing in TgCRND8 tissue coincides with decreased NOTCH3/JAG1 signalling, overproduction of endothelial filopodia and increased numbers of vascular pericytes. Using a novel in vitro approach to study sprouting angiogenesis in TgCRND8 organotypic brain slice cultures (OBSCs), we find that BACE1 inhibition normalises excessive endothelial filopodia formation and restores NOTCH3 signalling. These data present the first evidence for the potential of BACE1 inhibition as an effective therapeutic target for aberrant angiogenesis in AD.

## Introduction

Alzheimer’s disease (AD) is closely associated with alterations in the vascular system^1^. Multiple studies in humans and animal models have described pathological vascular changes in AD^2^, including disruption to the neurovascular unit^3^ and blood–brain barrier^4^, increased microvessel density^3,5,6^, arteriolar and venular tortuosity^7,8^ and vascular Aβ accumulation^9^. Such changes will likely compromise the effective delivery of oxygen and nutrients to the brain, so understanding whether vascular alterations are a cause or consequence of aspects of AD pathology, notably Aβ accumulation, is required in order to design effective therapies.

Amyloid beta peptides, particularly Aβ_1–42_, are hallmarks of AD^10^. These peptides are the result of sequential proteolytic cleavage of amyloid precursor protein (APP) by β- and γ-secretase enzyme activity. Whilst it is reported that synapse loss is the best correlate of clinical outcome in AD^11^, it is unclear as to whether pathological APP processing products drive this effect by a direct action on neurons, or indirectly such as through aberrant angiogenesis. Despite widespread interest in the role of brain vasculature in AD, little is known about how amyloid-induced vascular changes alter pathological sprouting angiogenesis.

Sprouting angiogenesis is responsible for the formation of new blood vessels in the cortex. This process (both pathological and physiological) encompasses sequential events including; sprouting at the vascular front of endothelial cells, extension of sprouts, the formation of new vascular loops and pericyte recruitment^12–14^. Pericyte recruitment is closely linked to endothelial cell sprouting. Endothelial tip cells secrete platelet derived growth factor (PDGF) that activates platelet derived growth factor receptor beta (PDGFRβ) on pericytes to induce their migration to the sprout^15,16^. Endothelial sprouting is regulated by the NOTCH signalling pathway^17^. NOTCH receptors (NOTCH1–4) undergo proteolytic processing via γ-secretase in a manner comparable to that of APP^18,19^ resulting in the hypothesis that interactions between these signalling pathways could underlie the angiogenic pathology in AD^20,21^.

Whilst indirect measures of angiogenic activity, such as vessel density, can be studied in post mortem brains, active processes, such as filopodia extension from tip cells, are notoriously difficult to observe in vivo. Mechanistic exploration of pathological angiogenesis using drugs, such as BACE1, is also complicated by issues of blood–brain barrier penetration and peripheral effects (such as metabolic changes) that may confound interpretation of results^22^. To permit mechanistic exploration of the relationship between amyloid pathology, NOTCH signalling and pathological angiogenesis, we used organotypic brain slice cultures (OBSCs), which provide an excellent experimental platform for such studies. OBSCs initially retain a dense network of capillaries and neurovascular units alongside maintenance of neuronal architecture and non-neuronal cell populations^23–25^. Crucially, OBSCs provide a three-dimensional culture system that supports the formation of new blood vessels and are amenable to pharmacological manipulation, live imaging and repeated measurements without interference from peripheral systems^26–29^. We and others have previously shown that OBSCs are powerful tools for investigating the progression of AD-like alterations including Aβ accumulation^30^, synaptic disruption^27,31^ and cerebrovascular damage^32^.

In this study, we find evidence for early pathological angiogenesis in the brains of postnatal TgCRND8 mice which was recapitulated in OBSCs. In addition to increased vessel density, OBSCs from TgCRND8 mice showed an increase in sprouting angiogenesis, that could be completely blocked by BACE1 inhibition. We investigate the mechanisms by which pathological APP processing and NOTCH signalling interact to induce excessive vascular sprouting and discuss the implications for the blood vessel pathology seen in AD.

## Results

### TgCRND8 mice show vascular abnormalities in the cortex

**Fig. 1:**
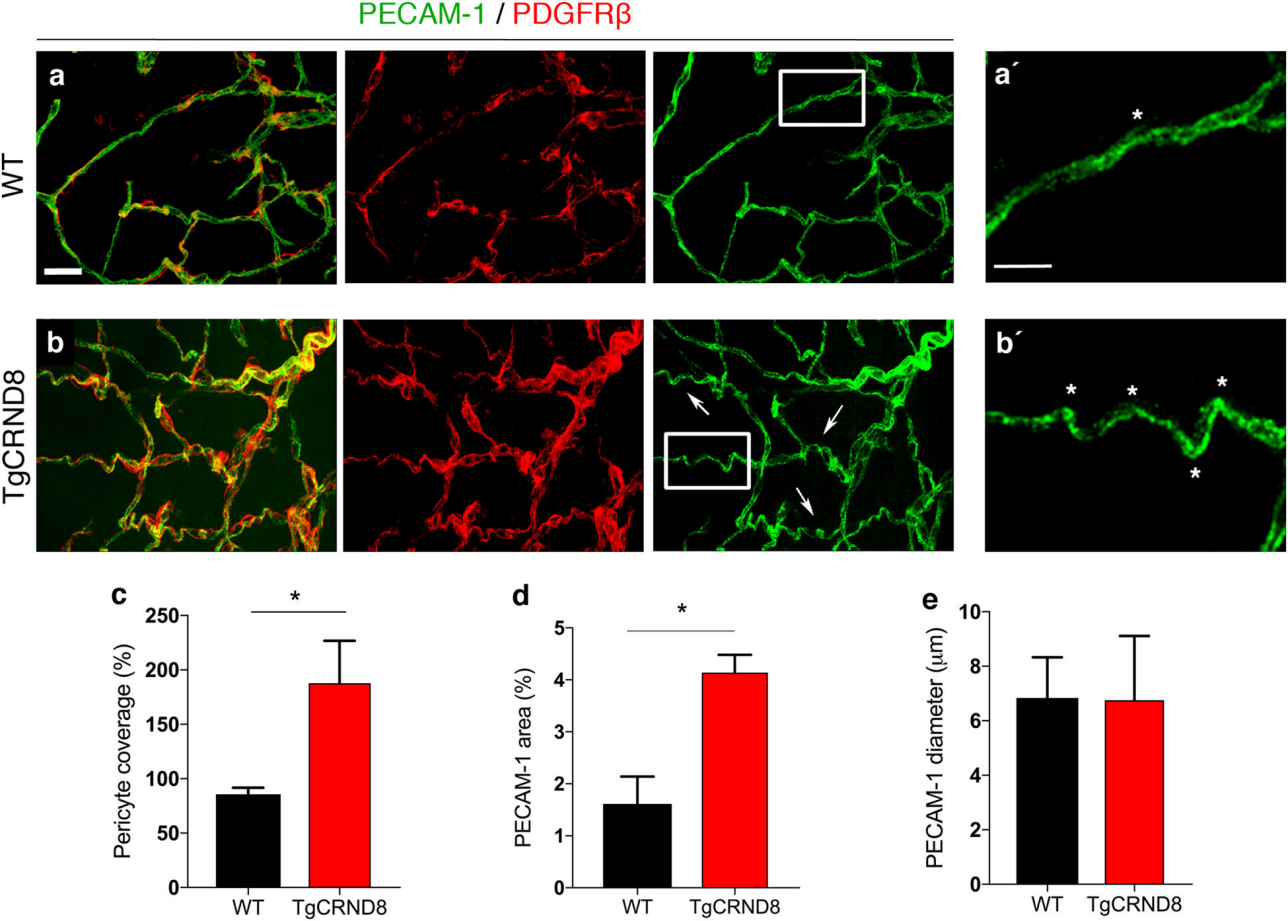
Vascular morphology in the cortex of postnatal TgCRND8 mice. **a**, **b** Blood vessels were stained by PECAM-1 and pericytes by PDGFRβ. Increased pericyte coverage and microvessel tortuosity (arrows and framed areas) is seen in P7 TgCRND8 cortex; scale bar 20 μm. (**a**′, **b**′) Enlarged white-framed areas in **a** and **b** showing samples of normal microvessels in WT (**a**′) and tortuous microvessels (*) in TgCRND8 (**b**′**)** cortex; scale bar 10 μm. **c** There is increased PDGFRβ^+^ pericyte coverage in TgCRND8 cortex (*p < 0.05). **d** There is an increase in PECAM-1^+^ area in TgCRND8 cortex (*p < 0.05). **e** No difference in vessel diameter was detected (*p* > 0.05); mean ± SD (*n* = 2 (WT), *n* = 4 (TgCRND8), Student’s *t*-test.

Vascular abnormalities have been widely reported in a range of amyloidosis mouse models, with conflicting reports demonstrating both increased and decreased microvessel density in adult transgenic mice^3,5,33^. Here, we sought to examine whether TgCRND8 mice show evidence of early pathological angiogenesis, as a potential reaction to the initiating steps of amyloidosis, where therapeutic intervention is more likely to be effective. We studied P7 TgCRND8 mouse post mortem cortex to determine whether a mutant huAPP transgene (and associated dysregulated amyloid processing), leads to pathological changes in the organisation of the vascular network and endothelial cell physiology during postnatal development (30,31) (Fig. 1). PECAM-1 (a marker of endothelial cells) expressing cortical blood vessels of P7 TgCRND8 mice (Fig. 1b, b′’) appeared to be more tortuous than cortical vessels of P7 WT mice (Fig. 1a, a′). When assessing PDGFRβ^+^ pericyte coverage of blood vessels in P7 TgCRND8 cortex, we found an ~2-fold higher pericyte coverage when compared to WT (Fig. 1c) with coverage becoming almost complete (Fig. 1b). We also found a significant increase in PECAM-1^+^ vessel density (Fig. 1d), with no significant alteration in vessel diameter (Fig. 1e).

### Characterisation of sprouting angiogenesis in organotypic brain slice cultures

**Fig. 2:**
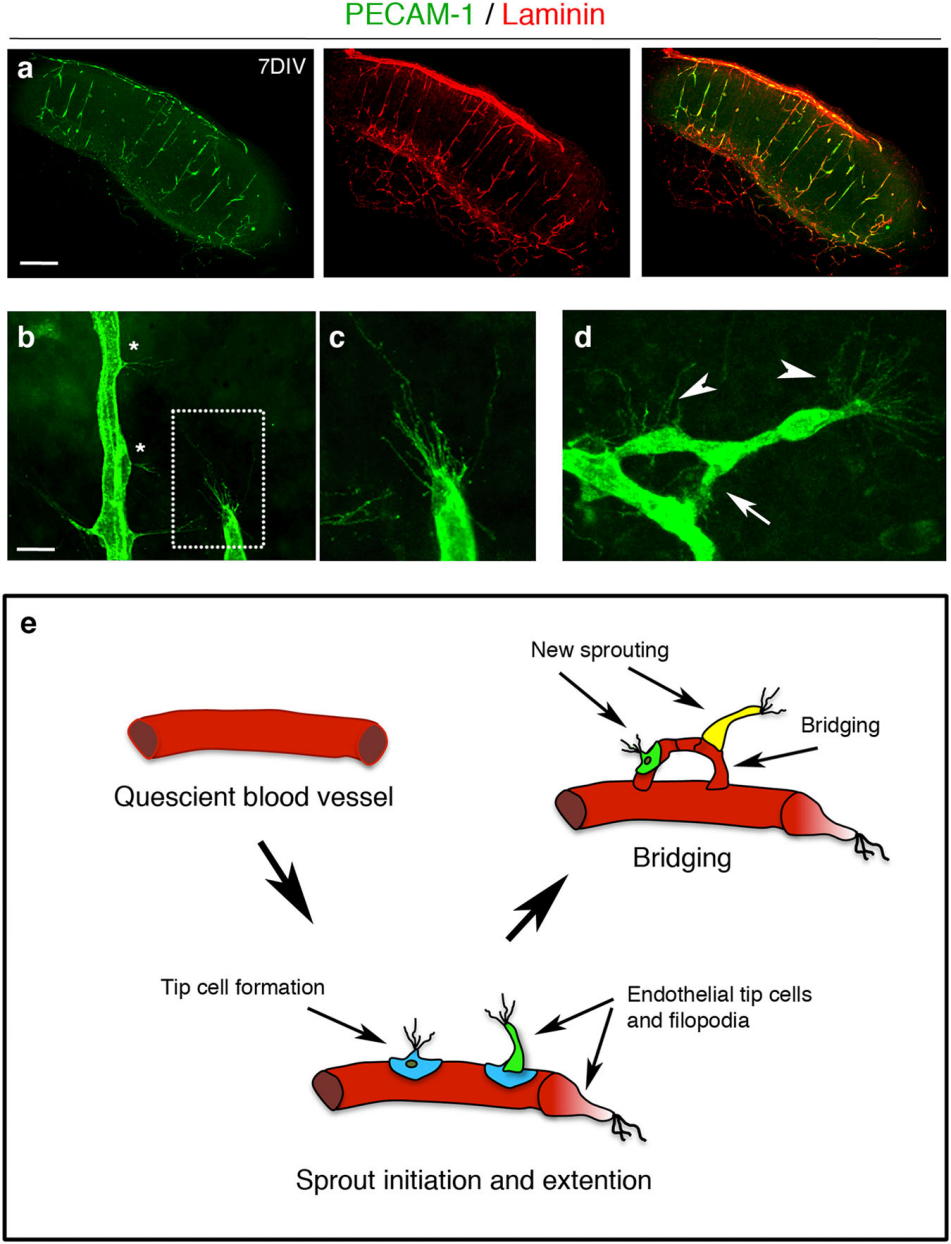
Characterisation of sprouting angiogenesis in 7-days in vitro organotypic cortical slice cultures from wild-type mice. **a** Confocal images of cortical slices stained for PECAM-1 (green) and laminin (red) to visualise blood vessels at 7 days in vitro, scale bar 500 μm. **b**–**d** Different stages of vascular sprouting can be visualised in cortical brain slices including; tip cell formation (*) endothelial tip extension (framed area in **b** which is expanded in **c**) new sprouting (arrow heads) and bridging (arrow) (scale bar 50 μm) (**d**). **e** Diagram summarising the different stages of sprouting angiogenesis that can be observed in cortical organotypic slice cultures.

We next sought to establish potential mechanisms by which the presence of a mutant huAPP transgene results in pathological angiogenesis. For this we required an experimental system in which key angiogenic processes could be easily observed and manipulated. We established an ex vivo cortical organotypic brain slice culture (OBSC) system to analyse this highly regulated process and found we were able to identify multiple stages of sprouting angiogenesis in OBSCs from wild-type (WT) mice (summarised in Fig. 2e) that were not observable in post mortem brain (Fig. 1). PECAM-1 staining showed a dense network of blood vessels expressing basal membrane protein laminin in 7-days in vitro cortical slices (Fig. 2a). Endothelial tip cells were found either along the trunk of PECAM-1^+^ blood vessels with few filopodia (Fig. 2b, asterisk) or at the leading edge of vascular sprouts extending numerous filopodia (Fig. 2b, c) indicating active sprouting angiogenesis. The filopodia of some vascular sprouts were also found to engage with those of a nearby endothelial tip cells to form a bridge and the formation of new blood vessels (Fig. 2d).

**Fig. 3:**
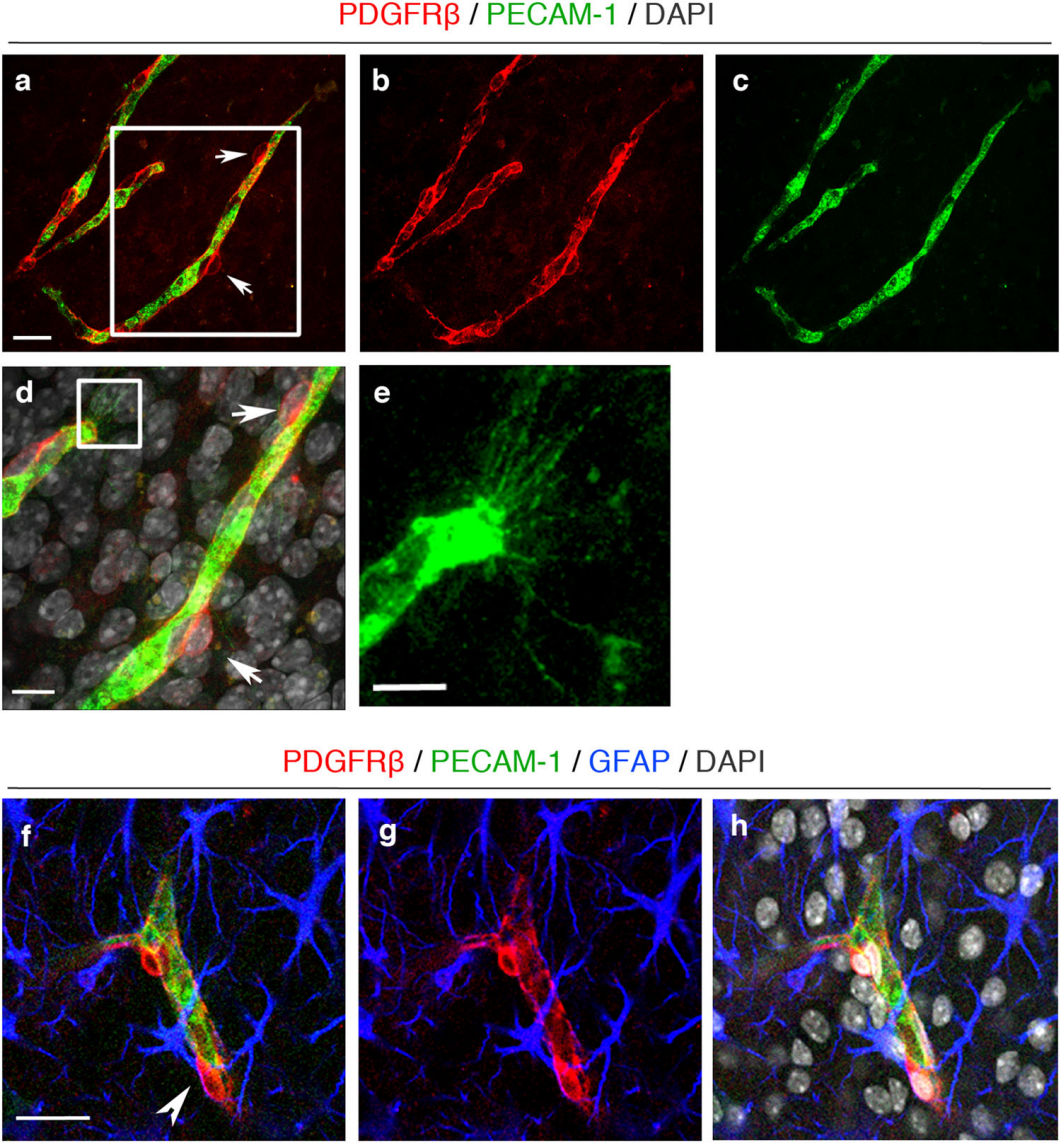
Pericytes are well preserved in organotypic cortical slices at 7 days in vitro. **a**–**c** Confocal images showing PECAM-1^+^ blood vessels (green) (**a**, **c**) covered by PDGFRβ^+^ pericytes (red, arrows (**a**, **b**)) in 7 days in vitro WT cortical slices; scale bar 20 μm. **d** Higher magnification images of the framed area in **a** with DAPI counterstain depicting nuclei (grey). Pericytes (arrows) can be seen on the PECAM-1^+^ angiogenic blood vessels; scale bar 10 μm. **e** depicts enlarged area framed in **d** showing clear vascular sprouting. **f**–**h** Confocal z-stacks show a preserved cytoarchitecture, consisting of PECAM-1^+^ blood vessels (green), surrounded by PDGFRβ^+^ pericytes (red) and GFAP^+^ astrocytes (blue) (DAPI = grey). Arrow marks the close association between the pericytes and astrocytes; scale bar 20 μm.

PDGFRβ^+^ pericytes were found around the OBSC blood vessels and with long cytoplasmic processes surrounding the abluminal surface of endothelium (Fig. 3a–c). High magnification confocal imaging showed that PDGFRβ expressing pericytes were closely associated with the PECAM-1^+^ vascular sprouts (Fig. 3d, e, framed image) and astrocytes within neurovascular units (Fig. 3f–h) at 7 days in vitro. Taken together, this demonstrates the utility of OBSCs as a tool to assess different steps of sprouting angiogenesis, including filopodia formation and pericyte coverage.

### Excessive vascular sprouting is associated with increased vascular density and number of pericytes in TgCRND8 OBSCs

**Fig. 4:**
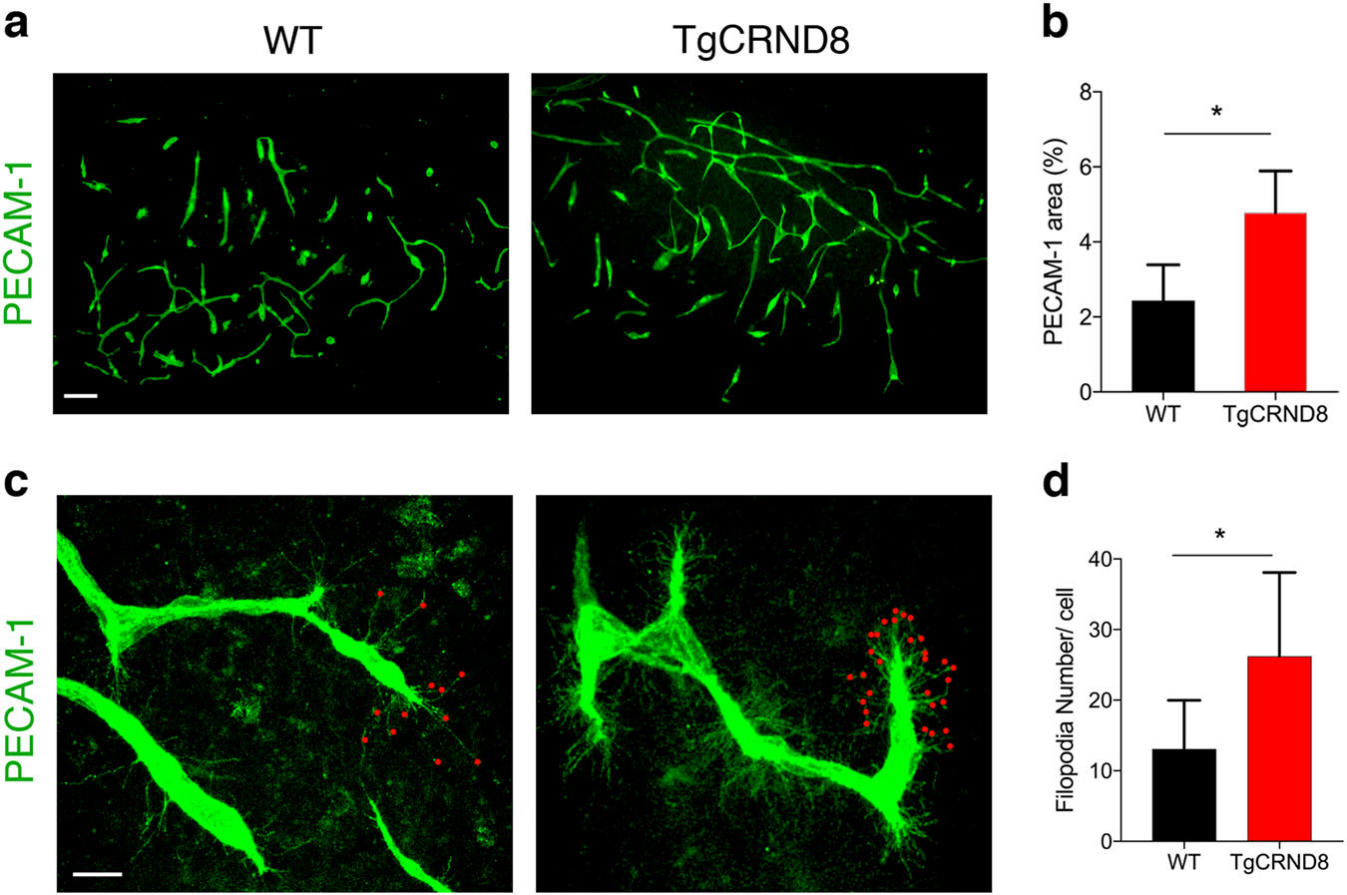
TgCRND8 organotypic cortical slices show increased vascular density and excessive filopodia formation when compared to wild-type cultures. **a** Representative confocal images showing blood vessel density (PECAM-1, green) in 7 days in vitro WT and TgCRND8 slices; scale bar 100 μm. **b** Quantification of PECAM-1^+^ area (as percentage of the total image) reveals a significantly higher blood vessel density in TgCRND8 cortical slices vs. WT slices. (mean ± SD (*n* = 5 (WT), *n* = 4 (TgCRND8), *P < 0.05 Student’s *t*-test). **c** Confocal images showing endothelial cells extending numerous finger-like filopodia at the forefront of vascular sprouts in 7 days in vitro WT and TgCRND8 cortical slices, visualised by PECAM-1 labelling. Red dots highlight vascular sprouting tips, scale bar 20 μm. **d** Quantification shows that the number of filopodia per cell is significantly higher in TgCRND8 when compared to WT slices. (mean ± SD, (*n* = 14 (WT), *n* = 13 (TgCRND8), *P < 0.05 Student’s *t*-test).

To determine whether OBSCs recapitulated our in vivo observations, we next looked for signs of vascular abnormalities in 7 days in vitro TgCRND8 OBSCs (Fig. 4). The capillary density (as measured by area of PECAM-1 staining) was significantly higher in TgCRND8 OBSCs compared to cultures from WT littermates (Fig. 4a, b). PECAM-1^+^ cells were found to co-localise with Ki67 (a marker of cell division) (Supplementary Fig. 1), potentially indicating increased endothelial proliferation as a contributor to increased vascular density. Confocal microscopy also revealed an increase in the number of filopodia found both at the leading edge, as well as along the body, of the vascular sprouts in TgCRND8 cultures (Fig. 4c) alongside a 50% increase in the number of filopodia at the vascular sprout of the blood vessels (Fig. 4d). Interestingly, OBSCs from an alternative amyloidosis mouse model (**5xFAD**) showed similar changes, with a significant increase in PECAM-1^+^ vessel length and area (Supplementary Fig. 2a–c) and a substantial increase in filopodia number (Supplementary Fig. 2d, e) compared to WT littermate controls, indicative of conserved mechanisms between models.

**Fig. 5:**
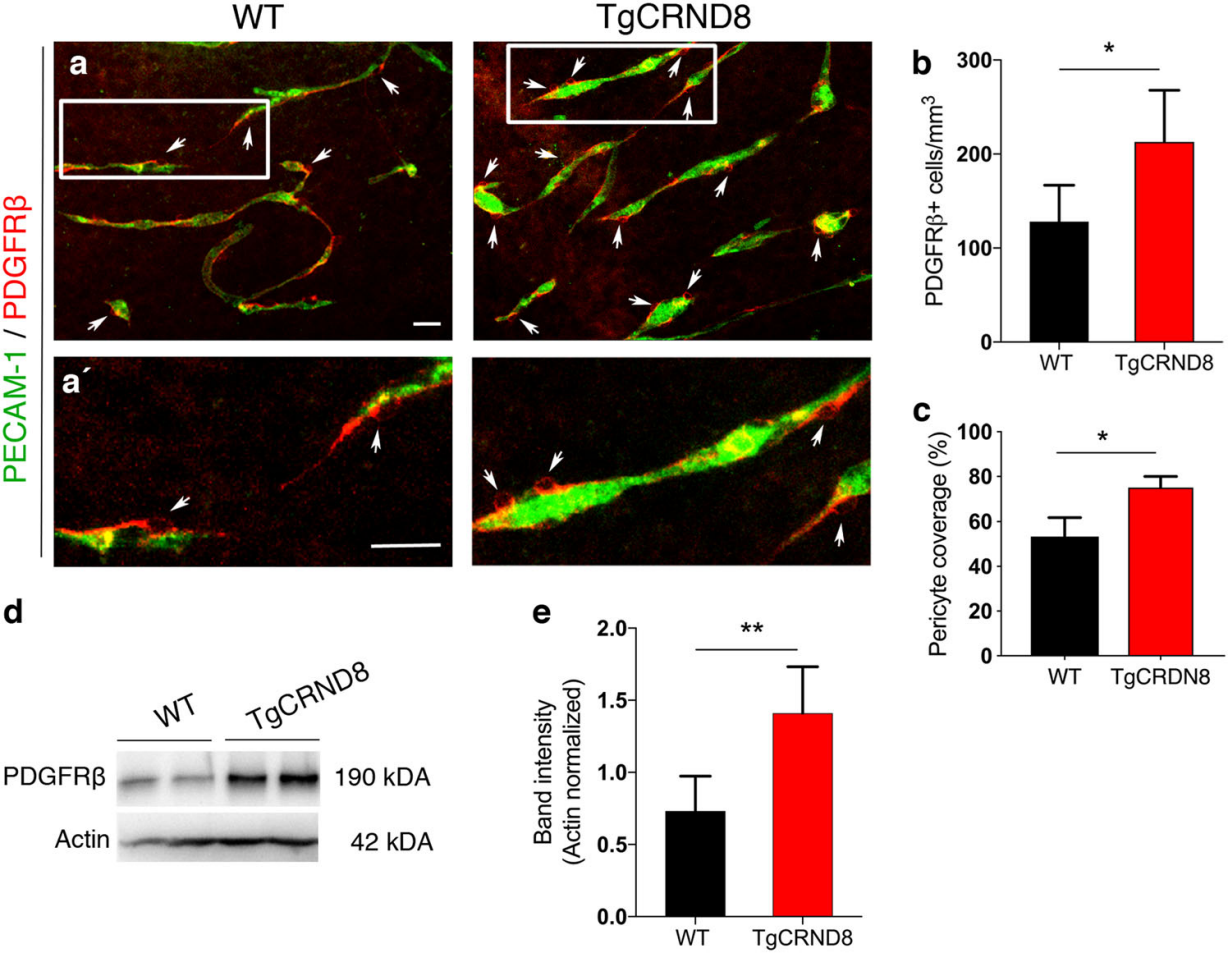
Increased pericyte number and PDGFRβ expression in TgCRND8 organotypic cortical slices. **a** Representative confocal images showing PDGFRβ^+^ pericytes (arrows) around blood vessels (PECAM-1, green) in 7 days in vitro WT and TgCRND8 slices; scale bar 50 μm. PDGFRβ^+^ pericytes are closely associated with cortical microvessels. **a**′ The framed areas in **a** are enlarged, scale bar 50 μm. **b** Quantification of PDGFRβ^+^ pericytes reveals an increased number in 7 days in vitro TgCRND8 slices when compared to WT (data are expressed as cell numbers per mm^3^. **c** Quantification of PDGFRβ^+^ staining area normalised to PECAM-1^+^ staining area shows a significant increase in pericyte coverage in TgCRND8 OBSCs; mean ± SD. (*n* = 4 (WT), *n* = 5 (TgCRND8), *P < 0.05 Student’s *t*-test). Representative western blots (**d**) and quantification of PDGFRβ band intensity (**e**) in 7 days in vitro WT and TgCRND8 cortical slices, shows increased PDGFRβ in TgCRND8 cultures when normalised to Actin control (*n* = 5 (WT), *n* = 4 (TgCRND8) **P < 0.01 Student’s *t*-test).

In 7 days in vitro TgCRND8 OBSCs, the number of PDGFRβ^+^ pericytes around the capillaries was significantly higher than WT controls (Fig. 5a, b) even when normalised to the increased density of blood vessels (Fig. 5c). The increased number of PDGFRβ^+^ pericytes was correlated with an upregulation of PDGFRβ protein expression as measured by western blot (Fig. 5d, e).

### Inhibition of BACE1 activity normalises vascular density and hypersprouting in TgCRND8 OBSCs

**Fig. 6:**
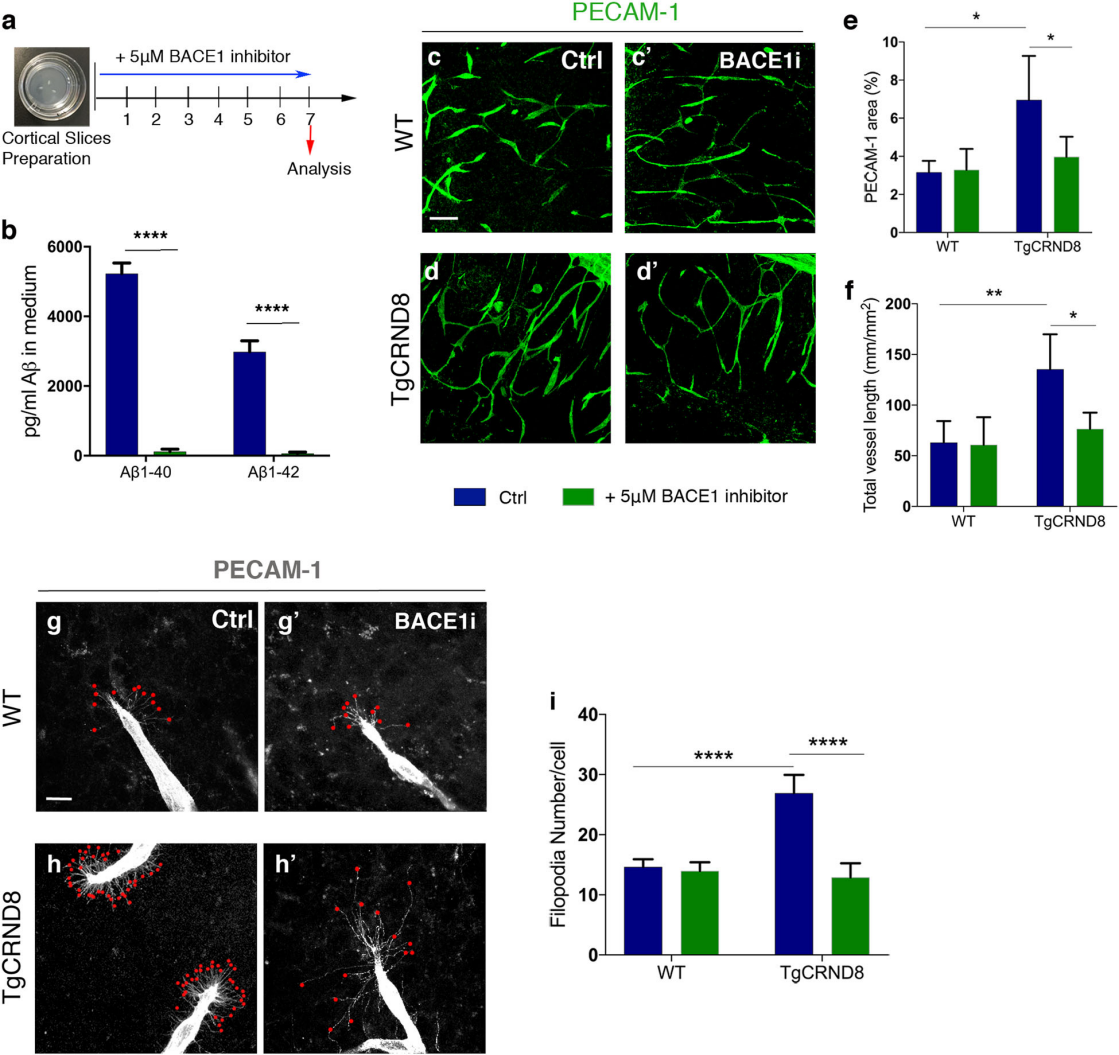
BACE1 inhibition decreases vascular density and normalises aberrant vascular sprouting in TgCRND8 organotypic cortical slices. **a** Diagram showing the experimental schedule for BACE1 inhibitor treatment of WT and TgCRND8 cortical slices. **b** Measurement of Aβ_1–40_ and Aβ_1–42_ in the culture medium of 7 day in vitro TgCRND8 slices treated with BACE1 inhibitor (mean ± SEM (*n* = 4), two-way ANOVA effect of treatment ****P < 0.0001). **c** Confocal images showing blood vessel density (PECAM-1, green) in 7 days in vitro control (**c**) and BACE1 inhibitor (**c**′) treated WT slices. **d**–**d**´ Confocal images showing blood vessel density (PECAM-1, green) in 7 days in vitro control (**d**) and BACE1 inhibitor (**d**′) treated TgCRND8 slices; scale bar 20 μm. BACE1 inhibition rescues the increase in PECAM1^+^ area (percentage image coverage) (mean ± SD (*n* = 4 (WT), *n* = 4 (TgCRND8), *P<0.05, two-way ANOVA) (**e**) and total vessel length (mm/mm^2^) (mean ± SD (*n* = 4 (WT), *n* = 4 (TgCRND8), **P<0.01 and *P < 0.05, two-way ANOVA) (**f**) seen in control treated TgCRND8 cultures. PECAM-1 staining of WT (**g**–**g**′) and TgCRND8 (**h**–**h**′) slice cultures with (**g′**, **h′**) or without (**g**, **h**) BACE1 inhibitor treatment. Individual filopodia are highlighted with red dots. **i** Quantification of the number of filopodia per cell demonstrates that BACE1 inhibitor rescues the increased number seen in untreated TgCRND8 cultures when compared to WT at 7 days in vitro (mean ± SD, (*n* = 4 (WT), *n* = 4 (TgCRND8), ****P<0.0001, two-way ANOVA).

To investigate if higher vascular density and filopodia number depend on the increased production of Aβ seen in TgCRND8 OBSCs, we applied the BACE1 inhibitor LY2886721 to OBSCs for 7 days in vitro (Fig. 6a). This inhibitor has been extensively characterised both in vivo and in vitro and found to be a potent and highly selective BACE inhibitor (with essentially no inhibition of cathepsin D, pepsin, renin or other similar aspartyl proteases)^34^. Using ELISA, we found that BACE1 inhibitor completely abolished the generation of Aβ_1–40_ and Aβ_1–42_ in the TgCRND8 OBSC culture medium (Fig. 6b) in agreement with previous characterisation of this inhibitor^34^. Interestingly, Aβ was found to co-localise with blood vessels in TgCRND8 OBSCs, but was absent in WT vessels (Supplementary Fig. 3a, b). Vascular Aβ was completely abolished by BACE1 inhibitor treatment (Supplementary Fig. 3b). BACE1 inhibition reduced the vascular density in TgCRND8 slices back to WT levels, with no additional effect on WT cultures (Fig. 6c–e). Quantification of PECAM-1^+^ capillaries revealed a twofold increase in total vessel length in TgCRND8 OBSCs, which was restored to WT levels after BACE1 inhibition (Fig. 6f). Similarly, BACE1 inhibition reduced the excessive sprouting activity of PECAM-1^+^ endothelial cells in TgCRND8 OBSCs, significantly lowering the number of filopodia at the leading edge of the vascular sprout back to WT levels (Fig. 6g–i). Conversely, direct application of synthetic huAβ_1–42_ to WT cultures resulted in a significant increase in PECAM-1^+^ vessel density and length (Supplementary Fig. 4a–d), further supporting a hypothesis whereby the overproduction of Aβ in TgCRND8 tissue can trigger pathological angiogenesis.

Despite significant rescue of hypersprouting (Fig. 6) we found that BACE1 inhibition did not normalise the increased levels of PDGFRβ in TgCRND8 OBSCs (Supplementary Fig. 5). Indeed, BACE1 inhibition increases the levels of PDGFRβ in WT OBSCs, but this is uncoupled from the lack of change seen in PECAM-1^+^ vessel coverage, length and filopodia number (Fig. 6).

### BACE1 inhibition restores *Notch3* and *Jag1* mRNA levels in TgCRND8 cortical slices

**Fig. 7:**
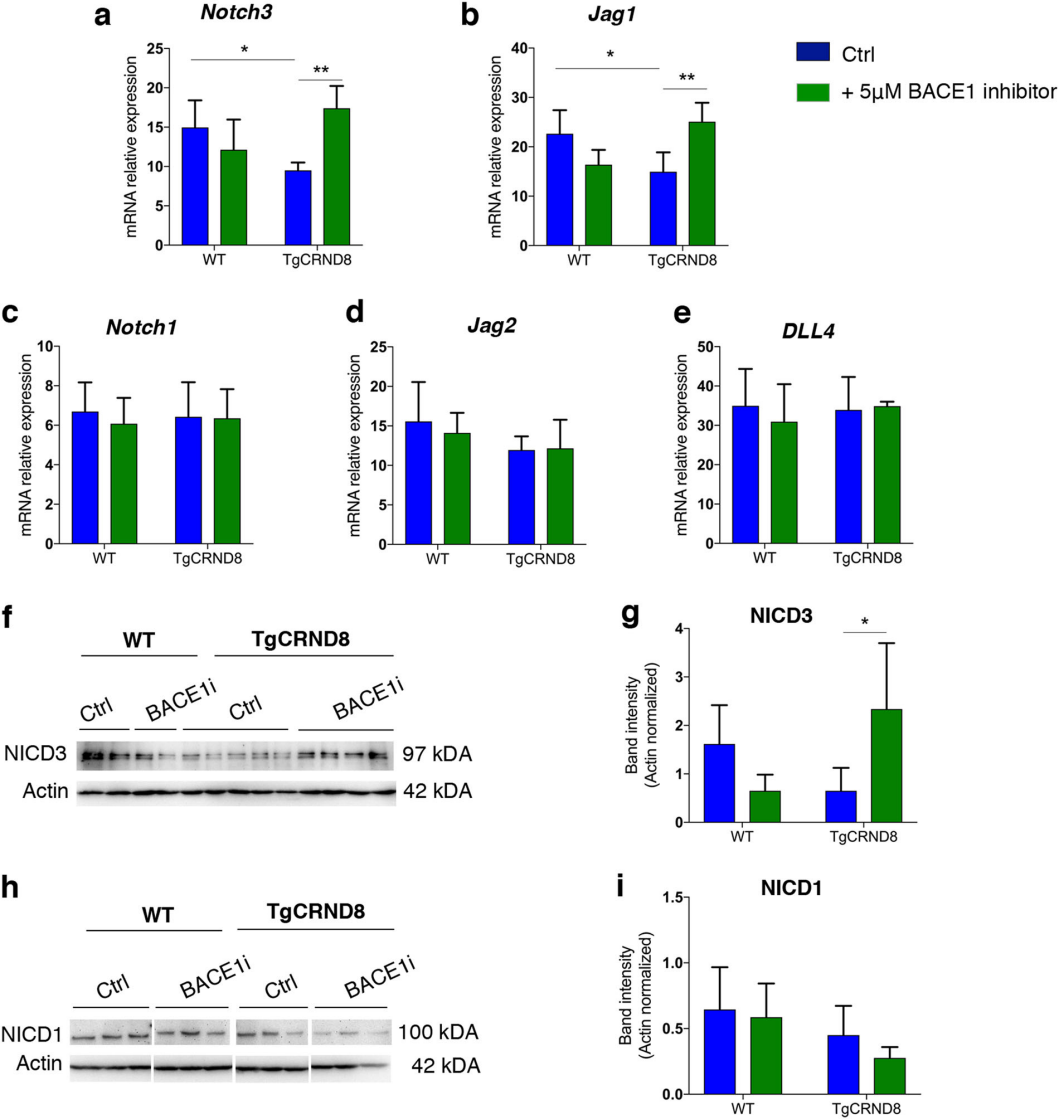
NOTCH signalling in BACE1-treated organotypic cortical slices. **a–e** Quantitative gene expression analysis of NOTCH receptors (*Notch1* and -*3*) and NOTCH ligands (*Dll4, Jag1, Jag2*) in 7 days in vitro WT and TgCRND8 OBSCs treated with BACE1 inhibitor. Untreated TgCRND8 OBSCs show a significant reduction in the levels of *Notch3* (**a**) and *Jag1* (**b**) compared to WT cultures. BACE1 inhibitor treatment normalised the expression levels of *Notch3* (**a**) and *Jag1* (**b**) in TgCRND8 cortical slices (mean ± SD, *n* = 5 (WT), *n* = 6 (TgCRND8), *P<0.05 and **P<0.01, two-way ANOVA). BACE1 inhibitor treatment had no effect on the expression of *Notch1* (**c**), *Jag2* (**d**) and *DLL4* (**e**) in 7 days in vitro TgCRND8 or WT cortical slices, (mean ± SD, *n* = 5 (WT), *n* = 6 (TgCRND8), *P* > 0.05, two-way ANOVA). **f**, **g** Representative western blots and quantification of NOTCH3 intracellular domain (NICD3) in 7 days in vitro WT and TgCRND8 cortical slices treated with BACE1 inhibitor. (Data expressed in band intensity; mean ± SD, *P<0.05, *n* = 4 (WT), *n* = 12 (TgCRND8), two-way ANOVA, Tukey post-hoc test.) **h**, **i** Representative western blots and quantification of Notch1 intracellular domain (NICD1) in 7 days in vitro WT and TgCRND8 cortical slices treated with BACE1 inhibitor (Data expressed in band intensity; mean ± SD, *n* = 9 (WT), *n* = 14 (TgCRND8), *P* > 0.05, two-way ANOVA).

Given that modulating APP/Aβ metabolism via BACE1 inhibition resulted in normalisation of hypersprouting, we hypothesised that interaction between Aβ peptide processing and NOTCH signalling might explain the endothelial hypersprouting observed in TgCRND8 mice. To test this hypothesis, we examined the mRNA levels of key components of the NOTCH signalling pathway, NOTCH1, NOTCH3, JAG1, JAG2 and DLL4, in control vs. BACE-inhibitor treated TgCRND8 and WT littermate OBSCs. Real-time quantitative PCR analysis showed that mRNA levels of *Notch3* (Fig. 7a) and *Jag1* (Fig. 7b) were significantly lower in TgCRND8 OBSCs when compared to the WT controls, whilst expression of *Notch1, Jag2* and *DLL4* were not significantly changed (Fig. 7c–e). In all, 5 μM BACEI inhibitor treatment for 7 days in vitro normalised both *Notch3* and *Jag1* mRNA expression back to the levels observed in WT cultures (Fig. 7a, b). We found no significant changes in the mRNA expression of *Notch1*, *Jag2*, *DLL4* in TgCRND8 or WT slices after BACE1 inhibitor treatment (Fig. 7c–e). Interestingly, application of synthetic Aβ to WT slices for 3 days in vitro resulted in a reduction in *Notch3* mRNA (Supplementary Fig. 4e) but did not alter the levels of *Jag1* mRNA (Supplementary Fig. 4f), potentially indicating that changes to *Notch3* are upstream to alterations in *Jag1*.

### BACE1 inhibition induces cleavage of NOTCH3 intracellular domain (NICD3) in TgCRND8 cortical slices

Finally, we analysed how higher production of Aβ affects NOTCH3 and NOTCH1 signalling activity in TgCRND8 OBSCs (Fig. 7f–i). Translocation of the NOTCH intracellular domain (NICD) into the nucleus is a negative regulator of endothelial sprouting^17^, so we tested whether the reduction in *Notch3* mRNA led to lower levels of NICD3. Western blot analysis showed a trend for reduced levels of NOTCH3 intracellular domain (NICD3) in TgCRND8 cortical slices (Fig. 7f, g). In contrast, BACE1 inhibitor treatment significantly increased NICD3 levels in TgCRND8 slices to at least the level of WT cortical cultures (Fig. 7f, g). Consistent with the mRNA levels of *Notch1*, there was no effect of genotype or BACE1 inhibition on the appearance of NOTCH1 intracellular domain (NICD1) (Fig. 7h, i).

## Discussion

In this study, we explored the relationship between Aβ processing, NOTCH signalling and pathological angiogenesis in cortical tissue from huAPP transgenic mice. We report that BACE1 inhibition can reverse the increased pathological angiogenesis observed in TgCRND8 OBSCs alongside restoration of NOTCH signalling. We find that pathological angiogenesis is an early event in TgCRND8 mice, and is detectable before amyloid plaque formation, synapse loss or cognitive deficits^35^. Restoration of physiological levels of angiogenesis early in the disease course may therefore be an important target for AD therapeutics.

This study took advantage of the OBSC system to explore mechanisms of pathological angiogenesis that are difficult to study in vivo. OBSCs are a potent, underused tool for exploring vascular phenotypes^23,36,37^ with few prior studies seeking to explore this in combination with AD models^32,38^. Interestingly, the increased sprouting angiogenesis we observed in TgCRND8 OBSCs is more pronounced than we find in vivo at a similar age. The OBSC method likely stresses the tissue via the slicing injury and/or the isolation from the systemic vasculature; stimulating sprouting angiogenesis. By allowing visualisation of such processes in both WT and TgCRND8 tissue, the OBSC system appears to unmask a mechanism that underlies the vascular changes in AD models that may otherwise be overlooked.

Our finding that tissue from postnatal TgCRND8 mice shows pathological angiogenesis is of interest in the context of aging being a major risk factor for AD. Studies examining human brain microvessel density in the context of “normal” ageing have revealed inconsistent results (increased/decreased/no change) in aged vs. young individuals depending on brain region and microvessel type studied^39–42^. Despite widespread interest in vascular pathology in AD, there is also debate as to the relative roles of pro- and anti- angiogenic processes during disease progression^1^. For example, whilst some studies demonstrate increased angiogenesis in post mortem human AD or huAPP-mouse brain^3,5,6,43^, others report endothelial cell apoptosis and loss of vasculature^33,44^. It seems likely that such studies represent different stages of disease, with initial increases in angiogenesis (potentially as a result of rising Aβ that occurs in ageing^45,46^) being overtaken by cell death in the end stages^1^. Clearly, more work is required to fully understand the complex relationships between age, AD and vascular pathology, although our work indicates pathological angiogenesis can be induced by dysregulated Aβ processing even in the absence of ageing.

A potential role for Aβ as a regulator of angiogenesis has been previously proposed from observations in both physiological and pathological conditions. In post mortem human AD brains, it has been shown that increased vascular density in the hippocampus^6^ and pericyte-mediated capillary restriction^47^ correlates with Aβ load. In amyloid mouse models, immunisation with Aβ peptides cleared plaques and restored capillary density to normal levels^48^ whilst inhibition of Aβ aggregation reduced arteriolar Aβ accumulation and tortuosity^7^. Application of synthetic Aβ to a number of models has also highlighted its pro-angiogenic role with increased endothelial cell proliferation, capillary bed density and vascular sprouting seen both in vitro and in vivo^20,49,50^. Our use of a novel OBSC platform that endogenously expresses mutant APP with all its processing products allows for careful exploration of the effects of Aβ as well as other APP-derived products on vascular pathology^51–53^. Our findings add weight to growing evidence that dysregulation of amyloid processing, or exogenously applied huAβ_1–42_ (Supplementary Fig. 4), can stimulate pathological angiogenesis. It is worth noting, that whilst we were unable to analyse the exact form (monomeric, oligomeric etc.) of Aβ present in the TgCRND8 cultures (due to the presence of serum in the culture medium and very low concentration in the slice tissue itself) we know from our previous work that the Aβ present, and thus likely responsible for our observed phenotypes, is soluble, thioflavin-S negative and is not sequestered in extracellular plaques^27^.

**Fig. 8:**
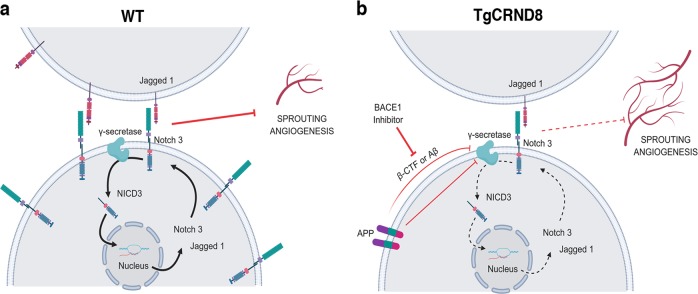
Proposed mechanism for the enhancement of sprouting angiogenesis by BACE1-dependent APP processing. Schematic diagram of our working hypothesis for increased sprouting angiogenesis in TgCRND8 (**b**) compared to WT (**a**) tissue. Increased APP processing by BACE1 in TgCRND8 OBSCs competes with NOTCH3 for γ -secretase or reduces γ-secretase activity, thereby lowering transcriptional signalling through NICD. This reduces *Notch3*-*Jag1* expression via autoregulatory mechanisms, thereby releasing the inhibitory influence on sprouting angiogenesis. Created with BioRender.

A key finding in this study is that overproduction of Aβ in TgCRND8 OBSCs is accompanied by reduced expression of the angiogenesis suppressor NOTCH3 and its ligand JAG1, providing a novel mechanistic insight into how amyloid pathology potentially impacts angiogenesis in AD. Previous studies have shown that *Notch3* knockout increases retinal vascular density and endothelial tip formation^54^ and silencing NOTCH3 in tumours promotes pathological angiogenesis^55^. NOTCH ligand JAG1 has also been implicated in angiogenic processes, with *Jag1* targeting antisense oligonucleotides potentiating FGF-responsive tube formation and invasion in vitro^56^. There are multiple potential mechanisms by which *Notch3* and *Jag1* could be downregulated in postnatal TgCRND8 tissue, which we summarise in our working hypothesis (Fig. 8).

NOTCH proteins and NOTCH ligands are substrates for the γ-secretase presenilin^57^, resulting in the production of NICD which translocates to the nucleus to regulate gene expression (Fig. 8a). Cleavage of NOTCH3 by γ-secretase has been found to induce *Notch3* and *Jag1* transcription via autoregulatory mechanisms^58^. Previous work has also shown that NOTCH3 activation (by cleavage to NICD3) is prevented by treatment with γ-secretase inhibitors^59^ which results in increased angiogenic sprouting^60^. Interestingly, this effect is mimicked by the application of synthetic monomeric Aβ potentially pointing to an enzymatic feedback inhibition, whereby high levels of Aβ lower the activity of γ-secretase^49^. This study aligns with our findings that application of synthetic Aβ to WT OBSCs results in increased microvessel density alongside a reduction in *Notch3* mRNA (Supplementary Fig. 4). In TgCRND8 tissue (Fig. 8b) increased levels of Aβ may act via this mechanism to inhibit the efficacy of γ-secretase, reducing levels of NOTCH3 cleavage and so lowering *Notch3* and *Jag1* transcription, ultimately resulting in increased sprouting angiogenesis. Alternatively, other APP processing products may also have inhibitory effects on γ-secretase. β-CTF, the result of BACE1 cleavage of APP, contains a region (Aβ_17–23_) that has been found to modulate γ-secretase activity by non-competitive inhibition^61^ and a similar role has been proposed for the APP intracellular domain (AID)^19^. Alternatively, increased expression of APP, or enhanced processing of APP through γ-secretase may directly compete with NOTCH ligands for enzymatic availability^62^.

In this study, we find that excessive endothelial filopodia formation and reduced NOTCH3/JAG1 signalling in TgCRND8 OBSCs can be normalised via application of BACE1 inhibitor (Fig. 8b) indicating a likely role for BACE1-dependent APP processing products. Interestingly, PDGFRβ protein remained elevated in BACE1-inhibitor treated TgCRND8 cultures and such treatment increased PDGFRβ in WT cultures (Supplementary Fig. 5), without impacting vascular phenotypes (Fig. 6). The role of pericytes in angiogenesis is dynamic and at times opposing^63^, with contrasting studies demonstrating their presence promotes early angiogenesis and vessel survival^13,64–66^, whilst inhibiting endothelial cell proliferation or inducing vessel regression in later stages^67–69^. This raises the possibility that increased pericyte expression could be a compensatory attempt at terminating pathological angiogenesis in TgCRND8 cultures which only becomes effective after normalisation of Aβ levels. BACE1 treatment in WT cultures may end ongoing physiological angiogenesis, resulting in the proliferation of pericytes on the stabilised, mature vessels. Alternatively, BACE1 may have an independent role in regulating PDGFRβ signalling that is uncoupled, at least in the short-term, from alterations in endothelial filopodia or microvessel density. BACE1 inhibitors have previously been shown to inhibit angiogenesis and tumour growth^70^. BACE1 knockout mice show reduced vascular density^71^ and treatment of zebrafish with BACE1 inhibitors was found to induce similar deficits, with application of Aβ restoring normal vascularisation^72^. Whilst we hypothesise that the normalisation of TgCRND8 angiogenesis after BACE1 inhibition is due to reduced levels of Aβ, we have to consider the possibility that BACE inhibition has a direct effect on angiogenic processes unrelated to APP. Other studies have found that BACE1 directly regulates JAG1 shedding, with BACE1^−/−^ mice showing an increase in *Jag1* levels and downstream NOTCH signalling^73^. This seems an unlikely mechanism in our system, however, due to the observation that BACE1 inhibition has no effect on the levels of *Jag1* (Fig. 7b) or vascular density (Fig. 6) in WT OBSCs and that application of synthetic Aβ alone increases PECAM-1^+^ vessel density (Supplementary Fig. 4). From our results, however, it is possible that BACE1 plays an independent role in pericyte recruitment/PDGFRβ expression, which warrants further exploration. Future studies could also examine whether the rescue effect of BACE1 inhibition on pathological angiogenesis is reversible, providing greater insight into the dynamics of angiogenesis in response to changes in amyloid processing.

Further mechanistic studies are needed to better understand the interplay between NOTCH signalling and APP processing mechanisms in AD. Here, we propose that APP overexpression or feedback inhibition from high Aβ/ β-CTF concentration in TgCRND8 OBSCs reduces γ-secretase-dependent cleavage of NOTCH3 (Fig. 8). Our results also indicate that there may be a safe level of BACE1 inhibition that can restore physiological levels of angiogenesis, without inducing angiogenic deficits in healthy tissue.

## Methods

### Mice

TgCRND8 mice^35^ were maintained on a mixed BL6:sv129 background. TgCRND8 mice overexpress human APP with both the Swedish (K670N/M671L) and Indiana (V717F) mutations. 5xFAD mice^74^ were maintained on a C57BL/6 x SJL background. 5xFAD mice express human APP with the Swedish (K670N/M671L), Florida (I716V) and London (V717I) mutations, alongside human PSEN1 with the M146L and L286V mutations. TgCRND8 or 5xFAD heterozygote males were bred with wild-type background matched females to produce both wild-type and transgenic heterozygote littermates. Animals were kept on a 12 h:12 h light:dark cycle at a constant temperature of 19 °C in a pathogen-free environment. All animal work was approved by the Babraham Institute Animal Welfare and Ethical Review Body, the University of Cambridge and the UK Home Office, and carried out in accordance with the Animals (Scientific Procedures) Act, 1986.

### Organotypic brain slice cultures

Cortical organotypic brain slice cultures were made from humanely killed P6–P9 littermates of either sex according to the method of de Simoni et al and our previous work^27,31,75^. Briefly, after schedule 1, brains were kept on ice in dissection buffer (EBSS supplemented with 25 mM HEPES and 1x Penicillin/Streptomycin). In all, 350-μm-thick sagittal sections of cortex were cut using a Leica VT1000S vibratome and slices collected using a modified sterile 3 ml Pasteur pipette. On average 6 cortical slices were collected per pup and stored in dissection buffer on ice until plating. For long-term culture, slices were transferred, in a class II hood, onto sterile Millicell® membrane inserts (Millipore PICM0RG50) in 35 mm culture dishes (Nunc). 3 cortical slices from the same pup were plated to a single membrane, with two dishes made per animal. Inserts were maintained in 1 ml of maintenance medium according to the following recipe: 50% MEM with Glutamax-1 (Life Technologies 42360–024), 25% Heat-inactivated horse serum (Life Technologies: 26050–070), 23% EBSS (Life Technologies: 24010–043), 0.65% D-Glucose (Sigma:G8270), 2% Penicillin/Streptomycin (Life Technologies 15140–122) and 6 units/ml Nystatin (Sigma N1638). Membrane inserts were handled with sterile forceps and the medium was changed 100% 4 h after plating and at 4 div. OBSCs were maintained at 37 °C, 5% CO_2_ and high humidity for 7 div. For BACE1 inhibition experiments, 1 culture per pup was treated with 5 µM BACE1 inhibitor LY2886721 (Selleckchem S2156) (previously reported to have high potency and specificity both in vivo and in vitro^34^) and compared with a DMSO treated control from the same animal. Cultures were treated for the entire 7 days in vitro. Synthetic huAβ_1–42_ was prepared according to a previously published protocol^76^ (see supplementary methods).

### Immunohistochemistry

#### OBSCs

OBSCs were fixed for 20 min in 4% PFA in phosphate buffered saline (PBS), washed three times in PBS, then blocked for 1 h in PBS supplemented with 0.5% Triton X-100 and 3% normal Goat Serum (Sigma G9023). Slices were incubated at 4 °C in primary antibody (diluted in blocking solution) overnight. In order to detect PDGFRβ, heat-mediated antigen retrieval was performed in 10 mM citrate buffer (pH 6.0) for 40 min at 80 °C prior to primary antibody incubation. Slices were washed a further three times in 0.5% Triton-X100 in PBS (PBS-TX) then incubated with secondary antibodies (Life Technologies and Jackson) (1:500 dilution in blocking solution for 2 h at 4 °C). Three final PBS-TX washes were conducted before slices were mounted on slides and images captured using a Leica Confocal Microscope. Primary antibodies used: rabbit anti-PDGFRβ (28E1) (1:200, Cell Signalling, Cat. No: 3169S), rat anti-PECAM-1 (1:400, BD, Cat. No: 550274), rabbit anti-laminin (1:200, Sigma, Cat. No: L9393), Mouse MOAB-2 (pan Aβ) (1:1000, Merck-Millipore, Cat. No: MABN254) Rabbit Ki67 (1:1000, Abcam, Cat. No: ab15580) secondary staining was conducted using species-specific fluorophore-conjugated (Streptavidin Alexa 488, Molecular Probes; Cy3 or Cy5, Jackson,) or biotin-conjugated secondary antibodies (Jackson). DAPI (1 μg/mL, Sigma) was used to counterstain nuclei.

#### Cryosections of postnatal mouse brain

P7 WT and TgCRND8 pups, of either sex were culled via cervical dislocation followed by decapitation. Brains were removed, snap-frozen, and embedded in Tissue-Tek® OCT compound (Sakura Finetek, The Netherlands). Cryosections were cut at 20 μm thickness and fixed in either ice cold acetone or 2% PFA for 20 min and air-dried. Cryosections were then stained for PECAM-1 and PDGFRβ as described above.

### Microscopy and image analysis

Images were captured via an epifluorescence microscopy system (Leica DM6000B) or using confocal microscopes (Leica, Zeiss LSM780). Figures were composed using Photoshop CS5 software. All analysis was done blind to genotype and/or treatment condition.

#### Quantification of pericyte number and coverage

To quantify the number of PDGFRβ-positive pericytes, cells were counted using NIH Image J Cell Counter tool. A maximum projection of fifteen-micrometre z-stacks was acquired from cortex slices freshly derived from WT and TgCRND8. Three pictures were taken at 40x for each slice. The areas of PDGFRβ^+^ pericytes and PECAM-1^+^ blood vessels were separately subjected to threshold processing and the respective signals for each image was calculated using NIH Image J Area Measurement tool. Pericyte coverage was determined as a percentage (%) of PDGFRβ^+^ pericyte area covering PECAM-1^+^ capillary surface area per field (ROI) 733 × 733 μm. Three slices per animal were analysed.

#### Capillary density, length and filopodia quantification

For PECAM-1^+^ capillary area, sections were analysed with Leica confocal microscope. Three pictures were taken at 20x for each section. ROI size of 733 × 733 μm for confocal images were utilised. The area covered by PECAM-1^+^ capillaries was analysed using the NIH ImageJ area measurement tool where pictures were subjected to threshold processing to produce a binary image. The area of PECAM-1 coverage was expressed as percentage of the total area, three slices per WT and TgCRND8 pups (*n* = 4–5 for each genotype) were analysed. The filopodia of vascular sprouts were analysed using z-stacked PECAM-1^+^ blood vessels.

### Protein extraction and western Blot

OBSCs were scraped off the membrane insert using a scalpel and transferred to 2x Laemelli buffer supplemented with 10% 2-mercaptethanol (50 µL per three slices). Samples were boiled for 10 min then frozen at −20 °C until use. Ten micrograms of protein were separated on a 10% SDS polyacrylamide gel then transferred onto PVDF membranes. Membranes underwent blocking (20 mM Tris, 136 mM NaCl, pH 7.6, 0.1% Tween 20 and 5% nonfat dry milk) before incubation with primary antibody anti-NOTCH1/NICD1 (1:750, Abcam, Cat No: ab3294), NOTCH3/NICD3 (1:1000, Abcam, Cat No: ab23426), PDGFRβ (28E1), (1:1000, Cell Signaling, Cat No: 3169 S), overnight at 4 °C. overnight at 4 °C. Signals were obtained by binding of a secondary anti-rabbit horse radish peroxidase (HRP) linked antibody (1:15000, Sigma-Aldrich, Cat No: A0545) and visualised by exposing the membrane to a charge-coupled device camera (LAS1000, Fujifilm, Tokyo, Japan) using a chemiluminescence kit (Merck Millipore, Billerica, MA, USA). Membranes were stripped and reprobed for β-actin (diluted 1:50 000, Sigma-Aldrich, Cat No: A3854). After densitometric analysis using Image J software, protein levels were calculated as percentage of β-actin expression.

### Aβ ELISA

Culture medium from 7 div OBSCs was assayed for human Aβ_1–40_ and Aβ_1–42_ using commercially available ELISA kits (Life Technologies: KHB3441 and KHB3481). Briefly, medium was incubated with Aβ detection antibody for 3 h, washed, and then incubated with an HRP-conjugated antibody for 30 min. After another wash step, stabilised chromogen was added for 30 min before the adding an acid-based stop solution. Absorbance was read at 450 nm using a PheraStar ELISA plate reader with the standard curve calculated using a 4-parameter fit. Concentration of Aβ in the medium is expressed as pg/ml. Levels of Aβ were compared between BACE1-treated and DMSO treated TgCRND8 cultures.

### Quantification of gene expression by qPCR

Slice cultures were scraped off the membrane and RNA extracted using an RNeasy mini kit (Qiagen). Briefly, three slices were homogenised in 350 µL lysis buffer RLT supplemented with 1% 2-mercaptethanol. In all, 350 μL 70% ethanol (in nuclease free water) was then added and samples transferred to RNeasy RNA collection columns. After several wash steps described in the kit protocol, the RNA was eluted in 20 µL nuclease free water, measured and quality tested using a Nanodrop® and frozen at −80 °C until use. cDNA synthesis was performed using Script™cDNA Synthesis Kit (Bio-Rad). cDNA was analysed using real-time PCR SsoAdvanced™ SYBR® Green Supermix from Bio-Rad and run on a Bio-Rad CFX96 real-time quantitative PCR (qPCR) system. Gene expression was normalised to the housekeeping gene GAPDH. Melt curve analyses were performed to ensure the specificity of qPCR product. Primer sequences are given in Supplementary Table 1. Values are presented as mean ± SD of three independent experiments, and within each experiment, triplicate samples were assessed.

### Statistical analysis

Experimental sample sizes were selected via power analysis of preliminary data using an online calculator: http://powerandsamplesize.com. All samples from the same animal, under the same treatment conditions were averaged to produce a single biological replicate. Each experiment was performed on 2–3 independent occasions. Statistical analysis was conducted using Graph Pad Prism. Data are expressed as mean ± SD. Two group comparison was performed by using Student’s *t*-test (two-sided) and multiple group comparison by one-way or two-way ANOVA followed by Tukey post-hoc test (to correct for multiple comparisons). For Western blot, after normalisation to the actin signal, the expression of each protein was compared using a two-way ANOVA, followed by Tukey post hoc test. Significance was set at *p* < 0.05.

## Supplementary information

Supplementary Methods and Figure Legends

Supplementary Figure 1

Supplementary Figure 2

Supplementary Figure 3

Supplementary Figure 4

Supplementary Figure 5

Supplementary Table 1

## Data Availability

The datasets generated and analysed in this study are available from the corresponding author on request.

## References

[1] Govindpani Karan, McNamara Laura G, Smith Nicholas R, Vinnakota Chitra, Waldvogel Henry J, Faull Richard LM, Kwakowsky Andrea (2019). Vascular Dysfunction in Alzheimer’s Disease: A Prelude to the Pathological Process or a Consequence of It?. Journal of Clinical Medicine.

[2] Jefferies WA (2013). Adjusting the compass: new insights into the role of angiogenesis in Alzheimer’s disease. Alzheimers Res. Ther..

[3] Giuliani A (2019). Age-related changes of the neurovascular unit in the cerebral cortex of alzheimer disease mouse models: a neuroanatomical and molecular study. J. Neuropathol. Exp. Neurol..

[4] Yamazaki Yu, Shinohara Mitsuru, Shinohara Motoko, Yamazaki Akari, Murray Melissa E, Liesinger Amanda M, Heckman Michael G, Lesser Elizabeth R, Parisi Joseph E, Petersen Ronald C, Dickson Dennis W, Kanekiyo Takahisa, Bu Guojun (2019). Selective loss of cortical endothelial tight junction proteins during Alzheimer’s disease progression. Brain.

[5] Biron KE, Dickstein DL, Gopaul R, Jefferies WA (2011). Amyloid triggers extensive cerebral angiogenesis causing blood brain barrier permeability and hypervascularity in Alzheimer’s disease. PloS ONE.

[6] Desai BS, Schneider JA, Li J-L, Carvey PM, Hendey B (2009). Evidence of angiogenic vessels in Alzheimer’s disease. J. Neural Transm..

[7] Dorr A (2012). Amyloid-β-dependent compromise of microvascular structure and function in a model of Alzheimer’s disease. Brain.

[8] Lai AY (2015). Venular degeneration leads to vascular dysfunction in a transgenic model of Alzheimer’s disease. Brain.

[9] Meyer EP, Ulmann-Schuler A, Staufenbiel M, Krucker T (2008). Altered morphology and 3D architecture of brain vasculature in a mouse model for Alzheimer’s disease. Proc. Natl Acad. Sci. USA.

[10] Hardy J (2002). The amyloid hypothesis of Alzheimer’s disease: progress and problems on the road to therapeutics. Science.

[11] Terry RD (1991). Physical basis of cognitive alterations in Alzheimer’s disease: synapse loss is the major correlate of cognitive impairment. Ann. Neurol..

[12] Chung AS, Ferrara N (2011). Developmental and pathological angiogenesis. Annu. Rev. Cell Dev. Biol..

[13] Ozerdem U, Stallcup WB (2003). Early contribution of pericytes to angiogenic sprouting and tube formation. Angiogenesis.

[14] Potente M, Gerhardt H, Carmeliet P (2011). Basic and therapeutic aspects of angiogenesis. Cell.

[15] Hellström M, Kalén M, Lindahl P, Abramsson A, Betsholtz C (1999). Role of PDGF-B and PDGFR-beta in recruitment of vascular smooth muscle cells and pericytes during embryonic blood vessel formation in the mouse. Development.

[16] Lindblom P (2003). Endothelial PDGF-B retention is required for proper investment of pericytes in the microvessel wall. Genes Dev..

[17] Phng L-K, Gerhardt H (2009). Angiogenesis: a team effort coordinated by notch. Dev. Cell.

[18] Hartmann D, Tournoy J, Saftig P, Annaert W, De Strooper B (2001). Implication of APP secretases in notch signaling. J. Mol. Neurosci..

[19] Roncarati R (2002). The gamma-secretase-generated intracellular domain of beta-amyloid precursor protein binds Numb and inhibits Notch signaling. Proc. Natl Acad. Sci. USA.

[20] Boscolo E (2007). β amyloid angiogenic activity in vitro and in vivo. Int. J. Mol. Med..

[21] Ethell DW (2010). An amyloid-notch hypothesis for Alzheimer’s disease. Neuroscientist.

[22] Lahiri DK, Maloney B, Long JM, Greig NH (2014). Lessons from a BACE inhibitor trial: off-site but not off base. Alzheimers Dement..

[23] Hutter-Schmid, B., Kniewallner, K. M. & Humpel, C. Organotypic brain slice cultures as a model to study angiogenesis of brain vessels. *Front. Cell Dev. Biol.* **3**, 52 (2015). 10.3389/fcell.2015.00052 PMC4557061 26389117

[24] Moser KV, Schmidt‐Kastner R, Hinterhuber H, Humpel C (2003). Brain capillaries and cholinergic neurons persist in organotypic brain slices in the absence of blood flow. Eur. J. Neurosci..

[25] Moser KV, Reindl M, Blasig I, Humpel C (2004). Brain capillary endothelial cells proliferate in response to NGF, express NGF receptors and secrete NGF after inflammation. Brain Res..

[26] Croft CL, Noble W (2018). Preparation of organotypic brain slice cultures for the study of Alzheimer’s disease. F1000Research.

[27] Harwell CS, Coleman MP (2016). Synaptophysin depletion and intraneuronal Aβ in organotypic hippocampal slice cultures from huAPP transgenic mice. Mol. Neurodegener..

[28] Holopainen IE (2005). Organotypic hippocampal slice cultures: a model system to study basic cellular and molecular mechanisms of neuronal cell death, neuroprotection, and synaptic plasticity. Neurochem. Res..

[29] Humpel C (2015). Organotypic vibrosections from whole brain adult Alzheimer mice (overexpressing amyloid-precursor-protein with the Swedish-Dutch-Iowa mutations) as a model to study clearance of beta-amyloid plaques. Front. Aging Neurosci..

[30] Novotny R (2016). Conversion of synthetic Aβ to in vivo active seeds and amyloid plaque formation in a hippocampal slice culture model. J. Neurosci..

[31] Sheppard O, Coleman MP, Durrant CS (2019). Lipopolysaccharide-induced neuroinflammation induces presynaptic disruption through a direct action on brain tissue involving microglia-derived interleukin 1 beta. J. Neuroinflammation.

[32] Kniewallner KM, Foidl BM, Humpel C (2018). Platelets isolated from an Alzheimer mouse damage healthy cortical vessels and cause inflammation in an organotypic ex vivo brain slice model. Sci. Rep..

[33] Religa P (2013). VEGF significantly restores impaired memory behavior in Alzheimer’s mice by improvement of vascular survival. Sci. Rep..

[34] May PC (2015). The potent BACE1 inhibitor LY2886721 elicits robust central Aβ pharmacodynamic responses in mice, dogs, and humans. J. Neurosci..

[35] Chishti MA (2001). Early-onset amyloid deposition and cognitive deficits in transgenic mice expressing a double mutant form of amyloid precursor protein 695. J. Biol. Chem..

[36] Chip, S., Zhu, X. & Kapfhammer, J. P. The analysis of neurovascular remodeling in entorhino-hippocampal organotypic slice cultures. *J. Vis. Exp.*10.3791/52023 (2014). 10.3791/52023 PMC4353386 25408363

[37] Ullrich C, Humpel C (2009). The pro-apoptotic substance thapsigargin selectively stimulates re-growth of brain capillaries. Curr. Neurovasc. Res..

[38] Daschil N (2015). L-type calcium channel blockers and substance P induce angiogenesis of cortical vessels associated with beta-amyloid plaques in an Alzheimer mouse model. Neurobiol. Aging.

[39] Sonntag, W. E., Eckman, D. M., Ingraham, J. & Riddle, D. R. in *Brain Aging: Models, Methods, and Mechanisms* (ed. Riddle, D. R.) (CRC Press/Taylor & Francis, 2007). 21204343

[40] Bell MA, Ball MJ (1981). Morphometric comparison of hippocampal microvasculature in ageing and demented people: diameters and densities. Acta Neuropathol..

[41] Hunziker O, Abdel’Al S, Schulz U (1979). The aging human cerebral cortex: a stereological characterization of changes in the capillary net. J. Gerontol..

[42] Kalaria RN (1996). Cerebral vessels in ageing and Alzheimer’s disease. Pharmacol. Ther..

[43] Thirumangalakudi L, Samany PG, Owoso A, Wiskar B, Grammas P (2006). Angiogenic proteins are expressed by brain blood vessels in Alzheimer’s disease. J. Alzheimers Dis..

[44] Fischer VW, Siddiqi A, Yusufaly Y (1990). Altered angioarchitecture in selected areas of brains with Alzheimer’s disease. Acta Neuropathol..

[45] Rodrigue KM (2012). β-Amyloid burden in healthy aging. Neurology.

[46] Rodrigue KM, Kennedy KM, Park DC (2009). Beta-amyloid deposition and the aging brain. Neuropsychol. Rev..

[47] Nortley R (2019). Amyloid β oligomers constrict human capillaries in Alzheimer’s disease via signaling to pericytes. Science.

[48] Biron KE, Dickstein DL, Gopaul R, Fenninger F, Jefferies WA (2013). Cessation of neoangiogenesis in Alzheimer’s disease follows amyloid-beta immunization. Sci. Rep..

[49] Cameron DJ (2012). Alzheimer’s-related peptide amyloid-β plays a conserved role in angiogenesis. PLoS ONE.

[50] Cunvong K, Huffmire D, Ethell DW, Cameron DJ (2013). Amyloid-β increases capillary bed density in the adult zebrafish retina. Invest. Ophthalmol. Vis. Sci..

[51] Moore S (2015). APP metabolism regulates tau proteostasis in human cerebral cortex neurons. Cell Rep..

[52] Walsh, D. M., Klyubin, I., Fadeeva, J. V., Rowan, M. J. & Selkoe, D. J. Amyloid-beta oligomers: their production, toxicity and therapeutic inhibition. *Biochem. Soc. Trans.* **30**, 552–7 (2002). 10.1042/bst0300552 12196135

[53] Willem M (2015). η-Secretase processing of APP inhibits neuronal activity in the hippocampus. Nature.

[54] Kofler NM, Cuervo H, Uh MK, Murtomäki A, Kitajewski J (2015). Combined deficiency of Notch1 and Notch3 causes pericyte dysfunction, models CADASIL and results in arteriovenous malformations. Sci. Rep..

[55] Lin S (2017). Non-canonical NOTCH3 signalling limits tumour angiogenesis. Nat. Commun..

[56] Zimrin AB (1996). An antisense oligonucleotide to the notch ligand jagged enhances fibroblast growth factor-induced angiogenesis in vitro. J. Biol. Chem..

[57] Groot AJ (2014). Regulated proteolysis of NOTCH2 and NOTCH3 receptors by ADAM10 and presenilins. Mol. Cell. Biol..

[58] Liu H, Kennard S, Lilly B (2009). NOTCH3 expression is induced in mural cells through an autoregulatory loop that requires endothelial-expressed JAGGED1. Circ. Res..

[59] Konishi J (2007). Gamma-secretase inhibitor prevents Notch3 activation and reduces proliferation in human lung cancers. Cancer Res..

[60] Kalén M (2011). Gamma-secretase inhibitor treatment promotes VEGF-A-driven blood vessel growth and vascular leakage but disrupts neovascular perfusion. PLOS ONE.

[61] Zhang Y, Xu H (2010). Substrate check of γ-secretase. Nat. Struct. Mol. Biol..

[62] Berezovska O (2001). Notch1 and amyloid precursor protein are competitive substrates for presenilin1-dependent γ-secretase cleavage. J. Biol. Chem..

[63] Winkler EA, Sagare AP, Zlokovic BV (2014). The pericyte: a forgotten cell type with important implications for Alzheimer’s disease?. Brain Pathol..

[64] Ribatti D, Nico B, Crivellato E (2011). The role of pericytes in angiogenesis. Int. J. Dev. Biol..

[65] Darland DC (2003). Pericyte production of cell-associated VEGF is differentiation-dependent and is associated with endothelial survival. Dev. Biol..

[66] Ozerdem U, Stallcup WB (2004). Pathological angiogenesis is reduced by targeting pericytes via the NG2 proteoglycan. Angiogenesis.

[67] Simonavicius N (2012). Pericytes promote selective vessel regression to regulate vascular patterning. Blood.

[68] Bergers G, Song S (2005). The role of pericytes in blood-vessel formation and maintenance. Neuro-Oncol..

[69] McIlroy M, O’Rourke M, McKeown SR, Hirst DG, Robson T (2006). Pericytes influence endothelial cell growth characteristics: Role of plasminogen activator inhibitor type 1 (PAI-1). Cardiovasc. Res..

[70] Paris D (2005). Inhibition of angiogenesis and tumor growth by β and γ-secretase inhibitors. Eur. J. Pharmacol..

[71] Cai J (2012). β-Secretase (BACE1) inhibition causes retinal pathology by vascular dysregulation and accumulation of age pigment. EMBO Mol. Med..

[72] Luna S, Cameron DJ, Ethell DW (2013). Amyloid-β and APP deficiencies cause severe cerebrovascular defects: important work for an old villain. PLoS ONE.

[73] Hu X, He W, Luo X, Tsubota KE, Yan R (2013). BACE1 regulates hippocampal astrogenesis via the Jagged1-Notch pathway. Cell Rep..

[74] Oakley H (2006). Intraneuronal β-amyloid aggregates, neurodegeneration, and neuron loss in transgenic mice with five familial Alzheimer’s disease mutations: potential factors in amyloid plaque formation. J. Neurosci..

[75] De Simoni A, MY, Yu L (2006). Preparation of organotypic hippocampal slice cultures: interface method. Nat. Protoc..

[76] Stine WB, Jungbauer L, Yu C, LaDu MJ (2011). Preparing synthetic Aβ in different aggregation states. Methods Mol. Biol..

