# Supplementary material for: Beta secretase 1-dependent amyloid precursor protein processing promotes excessive vascular sprouting through NOTCH3 signalling
Source: Cell Death Dis. 2020 Feb 6;11(2):98. doi: 10.1038/s41419-020-2288-4 (PMC7005019; doi:10.1038/s41419-020-2288-4)
Supplement: Supplementary file 1 — Supplementary Methods and Figure Legends [file 41419_2020_2288_MOESM1_ESM.docx]

**Supplementary Methods**

**Treatment of OBSCs with synthetic Aβ**

Synthetic Aβ was prepared according to previously published protocols for the production of monomeric Aβ ^76^. Briefly, 1mg of synthetic human Aβ^1-42^ (California Peptide Research, Inc) was dissolved in 222μl 1,1,1,3,3,3,-Hexafluro-2-Propanol (HFIP) and reconstituted for 30 minutes. 10μl aliquots were loaded into low-bind tubes and HFIP evaporated via running samples in a SpeedVac at room temperature for 10 minutes. The dried peptide films were stored at -20°C over desiccant until use. For monomeric Aβ, the peptide film was dissolved in 5μl DMSO to a final concentration of 2mM huAβ_1-42_ and left to reconstitute for 15 minutes before sonicating in a bath sonicator for 10 minutes. huAβ_1-42_ or DMSO-only control was then immediately added to freshly prepared wild-type OBSCs at a final concentration of 1μM for 3 days *in vitro* before slices were harvested for RNA analysis or fixed for quantification of PECAM-1^+^ blood vessels.

**Supplementary Figure Legends**

**Supplementary Figure 1. PECAM-1^+^ blood vessels co-express Ki67 in WT and TgCRND8 organotypic cortical slices**

Representative 3D confocal images showing PECAM-1^+^ endothelial cells that are positive for Ki67 (a proliferation marker) in 7 days *in vitro* WT (upper) and TgCRND8 (lower) OBSCs. Scale bar 20μm.

**Supplementary Figure 2. Increased vascular density and excessive filopodia formation in 5xFAD organotypic cortical slices**

**(a)** Representative confocal images showing blood vessel density (PECAM-1, green) in 7 days *in vitro* WT and 5xFAD slices; scale bar 50μm. **(b-c)** Quantification of PECAM-1^+^ vessel length **(b)** (mean ± SD (n=5 (WT), n=4 (5xFAD), *P<0.05 Student’s t-test) and PECAM-1^+^ vessel area (as % of the total image) **(c)** (mean ± SD (n=5 (WT), n=4 (5xFAD), ***P<0.001 Student’s t-test) reveals a significantly higher blood vessel density in 5xFAD cortical slices versus WT slices. **(d)** Confocal images showing PECAM-1^+^ endothelial cells extending numerous finger-like filopodia at the forefront of vascular sprouts in 7 days *in vitro* WT and 5xFAD cortical slices, scale bar 20μm. **(e)** Quantification shows that the number of filopodia per cell is significantly higher in 5xFAD when compared to WT slices. (mean ± SD, (n=11 (WT), n=17 (5xFAD), ****P<0.0001 Student’s t-test).

**Supplementary Figure 3. BACE1 inhibitor decreases Aβ expression around PECAM-1^+^ blood vessels in TgCRND8 organotypic cortical slices**

**(a)** Representative confocal images showing that there is no expression of Aβ in PECAM-1^+^ blood vessels in 7 days *in vitro* control **(upper panel)** and BACE1 inhibitor **(lower panel)** treated WT slices; scale bar 20μm. **(b)** Representative confocal images showing high expression of Aβ in PECAM-1^+^ blood vessels in control treated TgCRND8 slices **(upper panel)**, whereas BACE1 inhibition decreases Aβ expression in treated TgCRND8 **(lower panel)**; scale bar 20μm

**Supplementary Figure 4. Synthetic Aβ_1-42_ treatment increases vascular density and decreases NOTCH3 mRNA levels in WT organotypic cortical slices**

**(a)** Diagram showing the experimental schedule for Aβ treatment of WT cortical slices. **(b)** Confocal images showing blood vessel density (PECAM-1) in 3 days *in vitro* control **(left)** and huAβ_1-42_ **(right)** treated WT slices, scale bar 20μm. Aβ_1-42_ treatment increases total vessel length (mm/mm^2^) (mean ± SD (n=4 (Ctrl), n=4 (Aβ treated), **P<0.01, Student’s t-test) **(c)** and PECAM-1^+^ area (% image coverage) (mean ± SD (n=4 (Ctrl), n=4 (Aβ treated), *P<0.05, Student’s t-test) **(d)** when compared to control slices. **(e-f)** Quantitative gene expression analysis of a NOTCH receptor (*Notch3*) and NOTCH ligand *(Jag1)* in 3 days *in vitro* WT cortical slices treated with Aβ_1-42_. Aβ_1-42_ treatment decreases the expression levels of *Notch3* mRNA in WT cortical slices (mean ± SD (n=5 (Ctrl), n=5 (Aβ treated), *P<0.05, Student’s t-test) **(e)** but has no effect on the expression of *Jag1* mRNA (mean ± SD (n=5 (Ctrl), n=5 (Aβ treated), P>0.05, Student’s t-test) **(f).**

**Supplementary Figure 5. BACE1 inhibitor has no effect on PDGFRβ levels in TgCRND8 organotypic cortical slices.**

**(a)** Representative Western blots and quantification of PDGFRβ in 7 days *in vitro* WT and TgCRND8 cortical slices treated with BACE1 inhibitor**. (b)** BACE1 inhibitor increases PDGFRβ protein levels in 7 days *in vitro* WT cortical slices, however BACE1 inhibitor treatment had no effect on the expression of PDGFRβ in TgCRND8 slices. (Data expressed in band intensity; mean ± SD, **P<0.01, *P<0.05, n=6 (WT), n=6 (TgCRND8), two-way ANOVA, Tukey post hoc test.).

**Supplementary Table 1. Primer Sequences for qPCR experiments**
