# Supplementary figures and images for: Beta secretase 1-dependent amyloid precursor protein processing promotes excessive vascular sprouting through NOTCH3 signalling
Source: Cell Death Dis. 2020 Feb 6;11(2):98. doi: 10.1038/s41419-020-2288-4 (PMC7005019; doi:10.1038/s41419-020-2288-4)

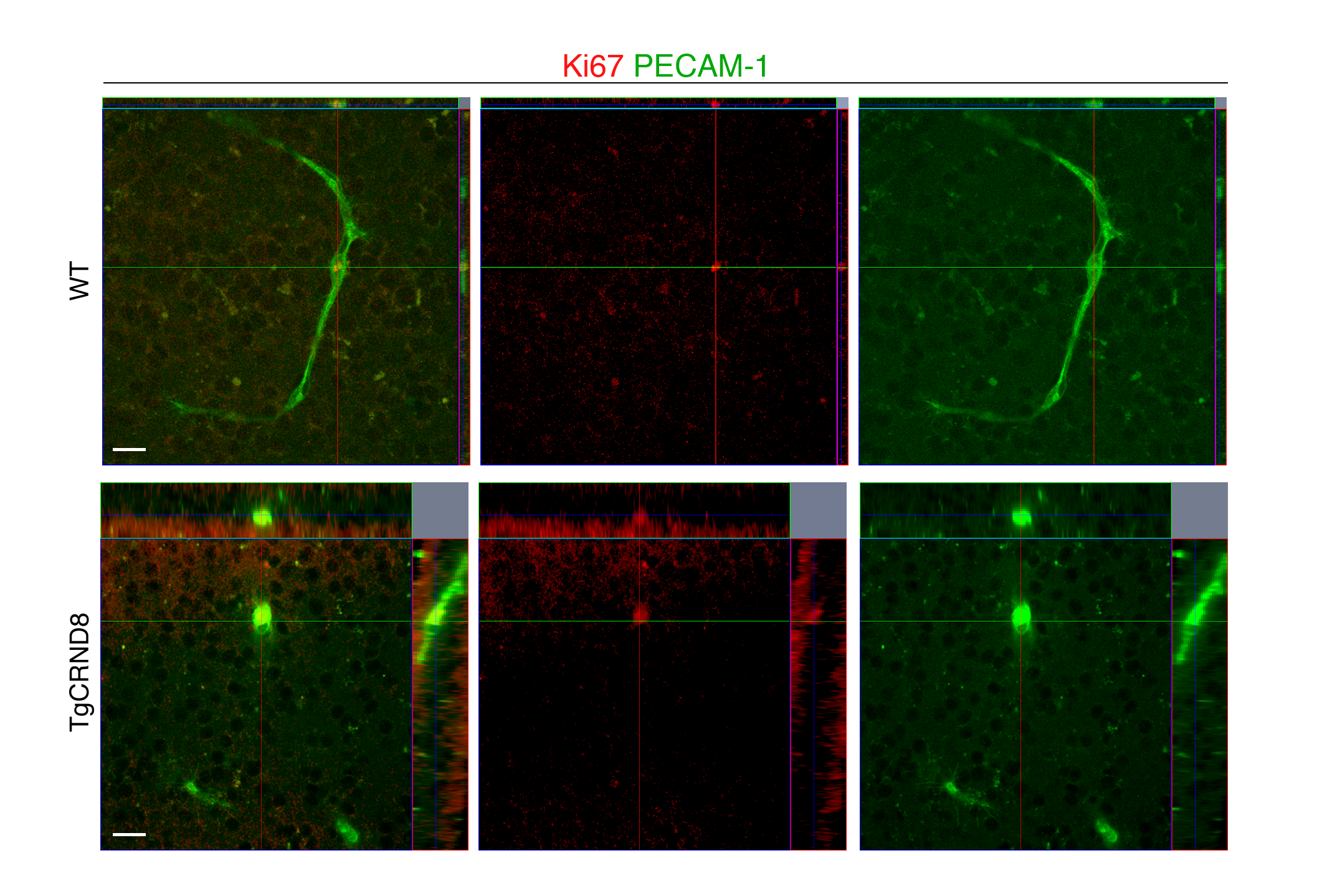

Supplement: Supplementary file 2 — Supplementary Figure 1 [file 41419_2020_2288_MOESM2_ESM.tif]

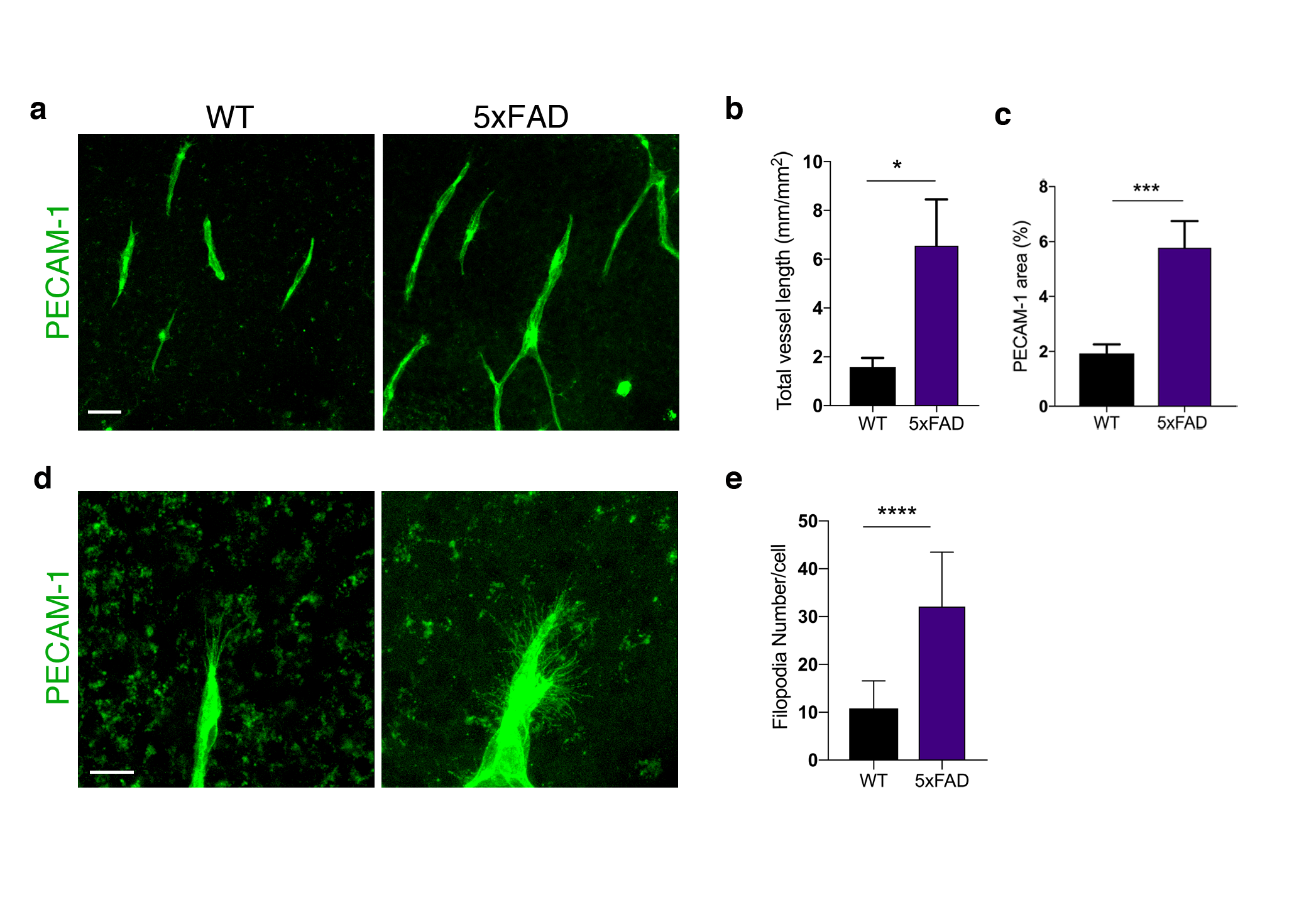

Supplement: Supplementary file 3 — Supplementary Figure 2 [file 41419_2020_2288_MOESM3_ESM.tif]

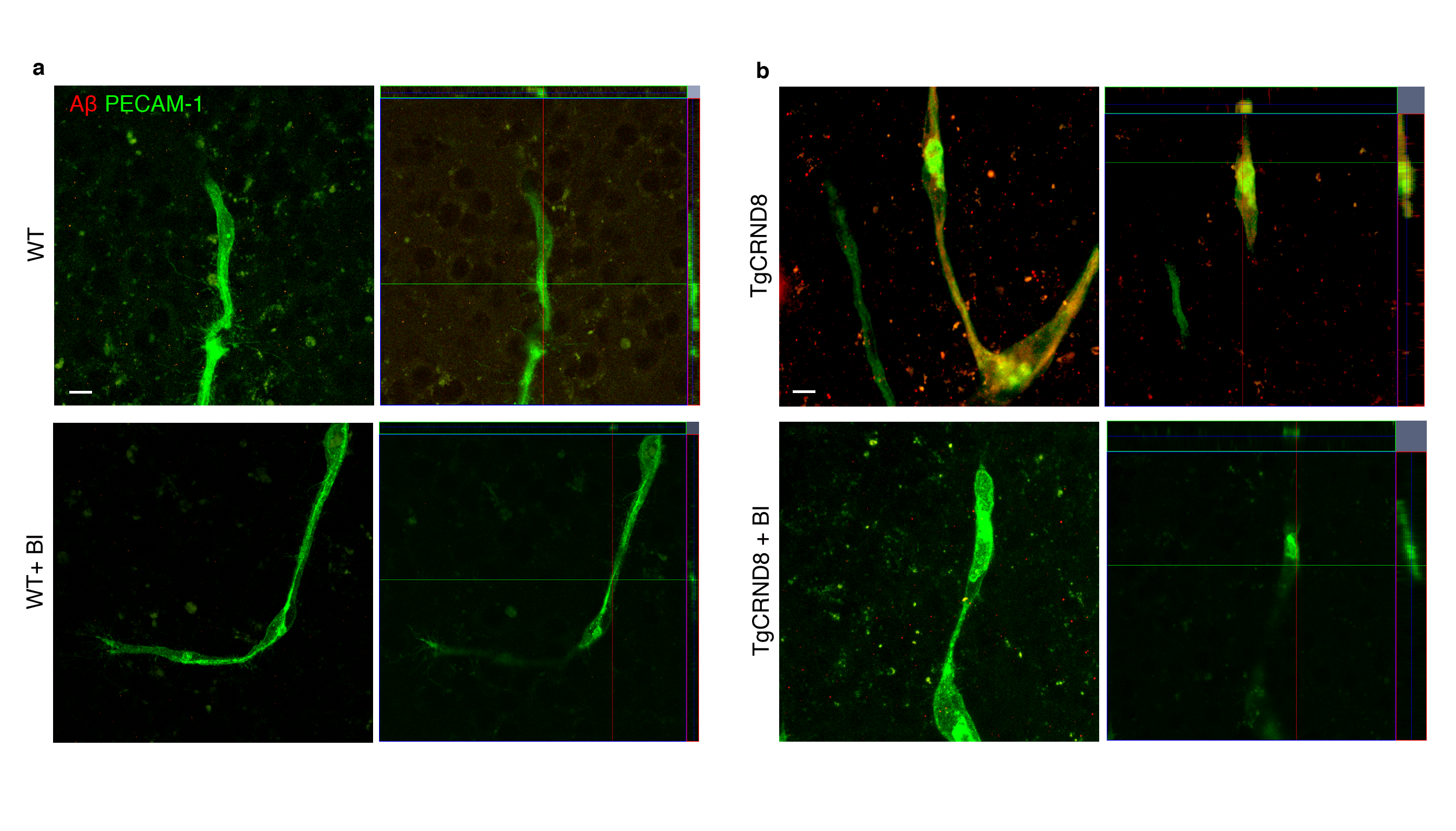

Supplement: Supplementary file 4 — Supplementary Figure 3 [file 41419_2020_2288_MOESM4_ESM.tif]

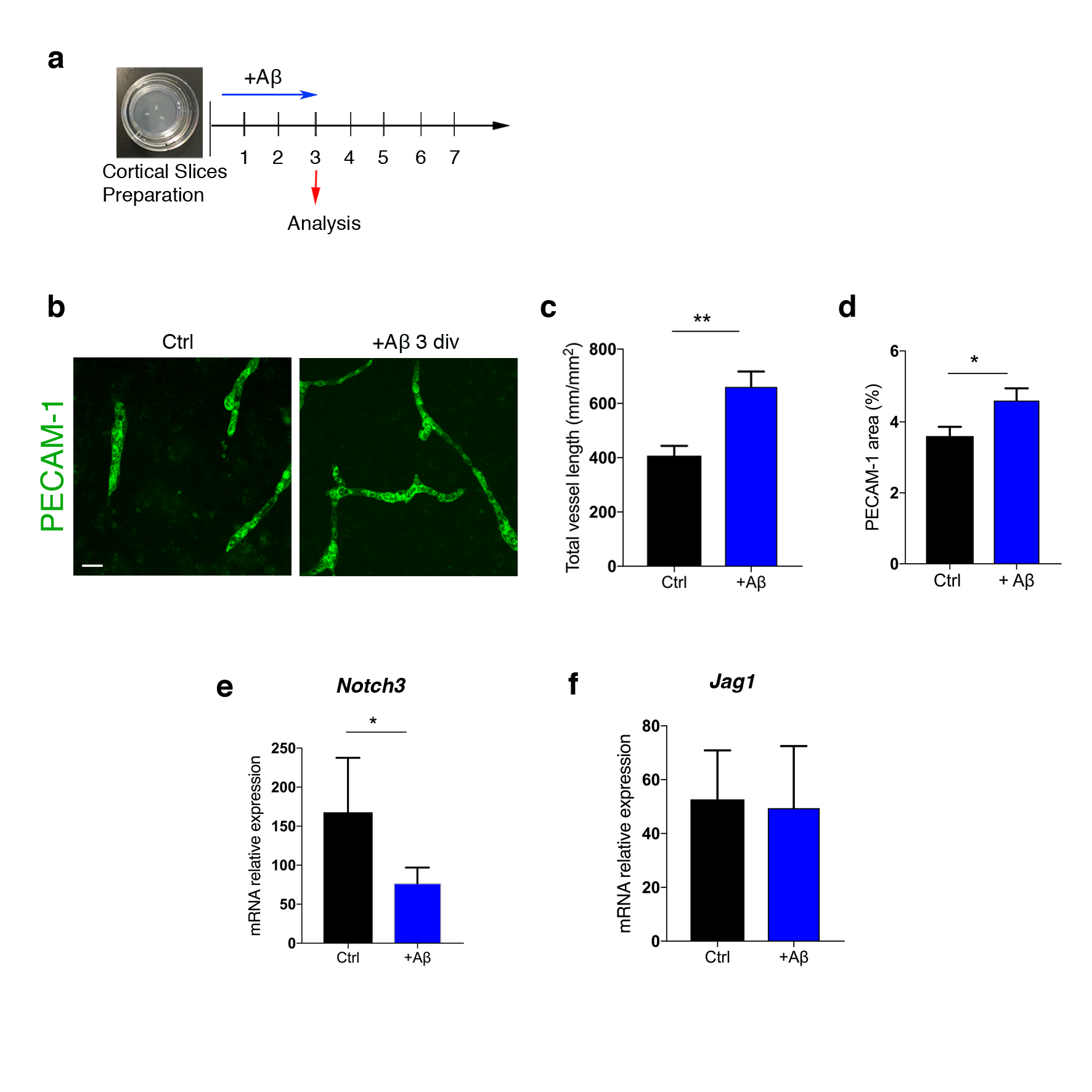

Supplement: Supplementary file 5 — Supplementary Figure 4 [file 41419_2020_2288_MOESM5_ESM.tif]

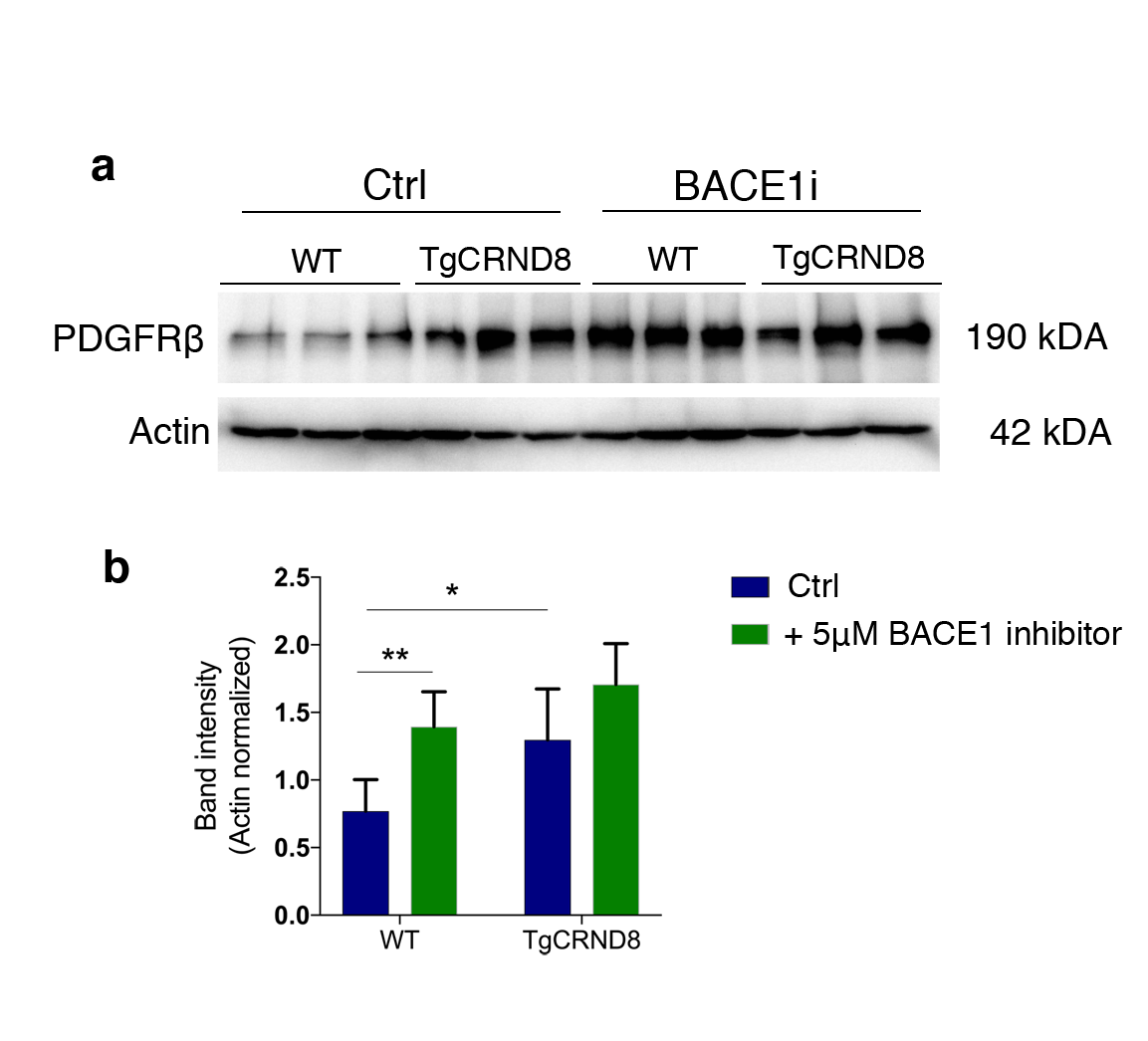

Supplement: Supplementary file 6 — Supplementary Figure 5 [file 41419_2020_2288_MOESM6_ESM.tif]
